# Occupational risk factors for meniscal lesions: a systematic review and meta-analysis

**DOI:** 10.1186/s12891-021-04900-7

**Published:** 2021-12-15

**Authors:** Carolin Bahns, Ulrich Bolm-Audorff, Andreas Seidler, Karla Romero Starke, Elke Ochsmann

**Affiliations:** 1 Luebeck Institute of Occupational Health (LIOH), University of Luebeck, Luebeck, Germany; 2 Department of Therapy Science I, Brandenburg Technical University Cottbus – Senftenberg, Senftenberg, Germany; 3 Division of Occupational Health, Department of Occupational Safety, Regional Government of South Hesse, Wiesbaden, Germany; 4 Associate Professor of Occupational Medicine, University Medical Center Giessen, Justus-Liebig-University, Giessen, Germany; 5 Institute and Policlinic of Occupational and Social Medicine (IPAS), Faculty of Medicine Carl Gustav Carus, Technische Universität Dresden, Dresden, Germany; 6 Institute of Sociology, Faculty of Behavioral and Social Sciences, Chemnitz University of Technology, Chemnitz, Germany

**Keywords:** Meniscus, Meniscal lesion, Occupation, Work, Systematic review, Meta-analysis

## Abstract

**Background:**

Meniscal lesions are common and are associated with the development of knee osteoarthritis. Knee-straining activities at work such as kneeling or squatting cause high biomechanical stresses on the knee joints that can lead to acute or chronic injuries. The objective of this systematic review is to update the evidence on the potential relationship between occupational risk factors and meniscal lesions.

**Methods:**

We searched the Medline, Embase and Web of Science databases until August 2021 to identify epidemiological observational studies on the association between occupational risk factors and meniscal lesions. Study selection, data extraction and risk of bias assessment were performed independently by two reviewers. Effect measures were extracted from individual studies and pooled with random effects meta-analysis. Heterogeneity analyses were conducted. We used GRADE (Grades of Recommendations, Assessment, Development and Evaluation) to assess the overall quality of evidence.

**Results:**

The database search resulted in 11,006 references, and 46 additional studies were identified through hand search. Twenty-two studies (represented in 25 publications) met the predefined eligibility criteria and nine records were included in the meta-analysis. There was only one study with an overall low risk of bias. Significant associations between occupational risk factors and the development of meniscal lesions were found for kneeling (effect size (ES) 2.15, 95% CI 1.67–2.76), squatting (ES 2.01, 95% CI 1.34–3.03), climbing stairs (ES 2.28, 95% CI 1.58–3.30), lifting and carrying weights ≥ 10 kg (ES 1.63, 95% CI 1.35–1.96), lifting and carrying weights ≥ 25 kg (ES 1.56, 95% CI 1.08–2.24), playing football on a professional level (ES 5.22, 95% CI 3.24–8.41), working as a hard coal miner (ES 5.23, 95% CI 2.16–12.69) and floor layers (ES 1.99, 95% CI 1.43–2.78). The overall quality of evidence according GRADE was moderate to low.

**Conclusion:**

We found consistent evidence of an increased risk of meniscal lesions by occupational knee-straining exposures. Our findings are important for the development of preventive strategies to reduce work-related knee disorders and work absence.

**Trial registration:**

PROSPERO (registration no. CRD42020196279).

**Supplementary Information:**

The online version contains supplementary material available at 10.1186/s12891-021-04900-7.

## Background

Meniscal lesions are common and knee meniscectomy is the most common procedure performed by orthopedic surgeons [1]. They are typically categorized as traumatic or non-traumatic based on their etiology. Traumatic meniscal lesions most commonly occur in younger active people and are caused by serious traumatic injury [2]. Non-traumatic lesions that result from repetitive stresses to the menisci over time often accompany knee osteoarthritis and are more common in middle-aged and older individuals [3, 4].

Meniscal lesions can lead to unspecific symptoms like pain and swelling accompanied by a locking or catching sensation in the knee [5]. However, structural damages in particular need not to correlate with the presence of pain [6] and often (52.1–78.1%) occur without symptoms [3, 7, 8]. Thus, they are challenging to assess, and incidence might be underreported. In a recent systematic review, Culvenor et al. [9] investigated the prevalence of meniscal damage in asymptomatic uninjured knees in adults based on magnetic resonance imaging (MRI) findings. The overall pooled prevalence of meniscal tears was 10%, with higher prevalence in individuals ≥ 40 years of age (19%). In this group, medial meniscal tears (14% (95% CI 8–20%)) were statistically significantly more common than lateral meniscal tears (5% (95% CI 2–8%)). The prevalence of meniscal injuries in asymptomatic athletes was even higher with changes of meniscal tissue in 31% [10].

There are indications that meniscal lesions are associated with the development of knee osteoarthritis [11, 12]. Total meniscectomy and partial lateral meniscectomy are risk factors for osteoarthritis of the knee [1]. There is some evidence that meniscus repair is associated with a lower risk for osteoarthritis of the knee compared with partial meniscectomy [13]. The risk of partial medial meniscectomy compared with conservative treatment for the future risk of osteoarthritis is not known. There is little research about risk factors for meniscal lesions. In a systematic review, Snoeker et al. [14] identified sex and age to be major risk factors for non-traumatic meniscal lesions and sports participation (playing rugby or football) to be associated with a high risk for acute meniscal tears. Further known risk factors for meniscal lesions are overweight, generalized joint hypermobility and time from anterior cruciate ligament (ACL) injury to reconstruction [14, 15]. High biomechanical stresses during knee-straining working positions may affect intra- and periarticular knee structures (e.g. cartilage, menisci, cruciate and collateral ligaments, bursae and patella tendon) and can lead to acute or chronic injuries [16, 17]. Snoeker et al. [14] indicated that there is also an association between occupational kneeling, squatting and frequent stair climbing and the development of meniscal lesions. However, these findings were based on only a few studies and no dose-response relationship was reported.

Meniscal lesions resulting from extended periods of work in a kneeling or squatting position are part of the European schedule of occupational diseases directly related to occupation [18]. They were accepted as occupational diseases in several EU member states. However, information regarding the required duration of exposure is rare.

Furthermore, identifying occupational risk factors is important in the development of prevention strategies for meniscal lesions at worksites. Therefore, a systematic review was conducted (1) to summarize the evidence on the potential relationship between occupational risk factors and the development of meniscal lesions, (2) to identify specific occupations or occupational activities at risk of meniscal lesions and (3) to assess whether a positive dose-response relationship is present.

## Methods

The study protocol was registered in the International prospective register of systematic reviews (PROSPERO) under record number CRD42020196279 and is available online at https://www.crd.york.ac.uk/prospero/display_record.php?ID=CRD42020196279. The systematic review with meta-analysis was performed in accordance with the criteria of the Preferred Reporting Items for Systematic Reviews and Meta-Analyses (PRISMA) statement [19] and the guidelines for conducting and reporting meta-analyses of observational studies in epidemiology (MOOSE) [20]. The systematic review also meets all criteria of AMSTAR 2 [21].

### Search strategy

We conducted a systematic literature search on Medline (via the Ovid interface), Embase (via the Elsevier interface) and Web of Science until 21th of August 2021 (search update; first search on 28th of February 2020). The research question was specified based on the Population, Intervention (Exposure), Control/Comparison, Outcome (PICO) scheme [22]. The search strategy combined a broad range of Medical Subject Headings (MeSH) and keywords describing the exposure (knee-loading exposure at work) and the outcome (meniscal lesion) to gain a highly sensitive search. Search terms for the exposure included knee-straining activities, occupations at risk for the development of knee disorders, and occupational determinants that were used in the search filter of Mattioli et al. [23]. No date or language restrictions were applied. A priori defined key articles [8, 24–27] identified through preliminary search for existing reviews were used to validate the search string. The search strategy was modified for each database accordingly and is described in Additional file 1. In addition, we conducted a manual search on grey literature (e.g. thesis, research reports, unpublished manuscripts) and used the “citation tracking function” by Web of Science to supplement the electronic search. Further, the reference lists of all included studies and related key reviews were reviewed manually.

### Eligibility criteria

We searched for epidemiological observational studies on the association between occupational risk factors and meniscal lesions. The following inclusion criteria were applied: (a) the study had a cohort, case-control, case-cohort or cross-sectional design with a response of at least 10%, (b) the study examined working population or retired workers (male and female, 16–75 years old), (c) the exposure was described as work-related knee-loading activities and positions or employments in specific occupational groups with intensive physical activities, (d) general population (16 years and older) or non-exposed workers served as comparison, (e) reported outcome was meniscal lesions diagnosed arthroscopically, by MRI, open surgery, clinical examination, diagnose codes (e.g. ICD-10) or self-reported. For the assessment of prevalence regarding meniscal lesions in specific occupational groups and exposure groups, cross-sectional studies without comparison group were included as well. Studies investigating injury-related meniscal lesions or secondary complaints after osteoarthritis or ACL-injury were excluded.

### Study selection

All citations were exported to EndNote X9.1 and duplicates were removed. Two reviewers (CB and UBA) independently screened the titles and abstracts of the remaining studies against the described in- and exclusion criteria. Subsequently, the same two reviewers checked full texts for eligibility. For excluded full text reports, the reasons for exclusion were recorded. Any disagreements during the selection process were resolved by discussion or, if needed, a third reviewer was consulted.

### Data extraction

From identified studies the following data were independently extracted by two reviewers (CB and UBA): study characteristics (authors, year of publication, country of origin, study design), study population (setting, sample size, demographics, response), occupational exposure (definition, job title, method used to identify the exposure), outcome (definition, assessment, localization of meniscal damage), and study results (number of participants analyzed, prevalence or incidence of the outcome in exposed and comparison subjects, relative risk measures, data indicating dose relationship, confounders). Discrepancies were resolved through discussion.

### Quality assessment

The same two reviewers (CB and UBA) independently assessed the risk of bias for each included study using a modified set of predefined criteria according to Ijaz et al. [28] and Kuijer et al. [29] (see Additional file 2). The following items were considered as major domains: (i) recruitment procedure and follow-up, (ii) exposure definition and measurement, (iii) outcome source and validation, (iv) confounding and effect modifications, (v) analysis method (methods to reduce research bias), (vi) chronology. The items (vii) blinding of assessors, (viii) funding and (ix) conflict of interest were considered as minor domains. Each item was categorized as either high risk, low risk or unclear risk of bias. Disagreements were discussed in consensus meetings moderated by the principal investigator. Studies were classified as low risk of bias if all major domains scored low risk. In other cases, studies were considered as high risk of bias.

### Data synthesis

Meta-analyses were conducted to pool the results from included studies regarding different occupational exposures as risk factors for the development of meniscal lesions. When available, we used the fully adjusted risk estimates of the individual studies. Unadjusted prevalence ratios were manually calculated if they were not reported in the studies, but the necessary information on frequency distributions was available. Because meniscal lesions are common in the general population [9] and odds ratios (ORs) tend to overestimate the relative risk when the prevalence of the outcome of interest is high, we converted the ORs to prevalence ratios for studies with a prevalence higher than 10%, according to the methods of Zhang and Yu [30]. The pooled risk of occupational exposure to meniscal lesions was estimated using a random effects model for the meta-analysis. If at least two primary studies which were comparable in terms of exposure and outcome were included, the meta-analysis was performed. The I^2^ value was used as a measure of heterogeneity. The occurrence of publication bias was determined using funnel plots and Egger’s tests, if at least three studies were included in the meta-analysis. Synthesis calculations were conducted using Stata Version 14.2 (StataCorp, College Station, TX).

Data from studies that were not eligible for meta-analysis was summarized qualitatively.

### Assessment of evidence

The Grading of Recommendations, Assessment, Development, and Evaluation (GRADE) approach [31] was used to assess the quality of the total body of evidence, following the example of Hulshof et al. [32] with modifications [33, 34]. We considered three levels of quality: high, moderate, and low, with an initial “high” level indicating the presence of randomized studies. However, as only observational studies were included, the starting level was set to “moderate”. The quality of evidence was downgraded based on five factors: quality of study limitations, indirectness, inconsistency, imprecision (range of the CI of studies > 2.0), and publication bias. An upgrade would follow if the study findings had large effect sizes (ES) (an effect estimate > 2.0), a dose-response relationship, and the presence of residual confounding (which would increase confidence in the association). If a pooled ES larger than 5.0 was present, the quality of evidence was upgraded twice.

## Results

### Literature search

**Fig. 1:**
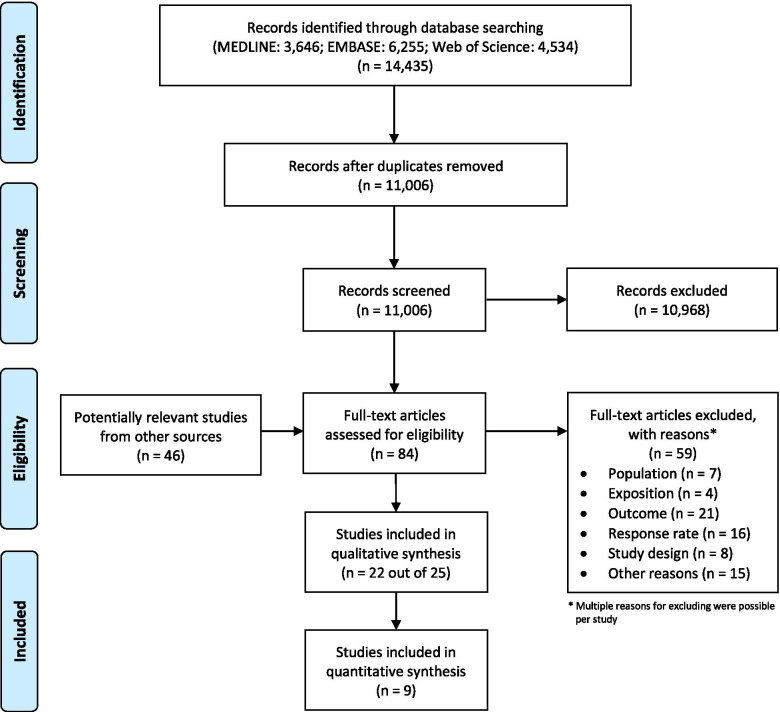
Modified PRISMA (Preferred Reporting Items for Systematic Reviews and Meta-Analyses) flow chart of the included studies

The literature search in Medline, Embase and Web of Science databases resulted in 14,435 records. After removal of duplicates, titles and abstracts of 11,006 articles were screened for eligibility. Full text assessment was performed on 84 studies, including 46 additional articles identified through citation tracking, screening of reference lists and grey literature. In total 25 studies [8, 17, 24–27, 35–53] met the inclusion criteria. The articles by Gotthardt et al. [51] and Gotthardt [52], as well as Rytter et al. [8], Jensen et al. [48] and Jensen et al. [49] were regarded as one in the analysis, respectively, because they involved the same study population, resulting in 22 studies included in this review. Of these, nine studies were eligible for meta-analysis. The detailed study selection process is summarized in Fig. 1. A list of the excluded studies and the reasons for exclusion is displayed in Additional file 3.

### Study characteristics

Of the 22 included records, 16 studies had a cross-sectional design, four were case-control studies [24–26, 51] and two cohort studies [35, 36]. Two studies were from the United States [40, 50] and one from South Korea [53] but the majority was conducted in Europe: Germany (*n* = 6, Federal Republic of Germany (*n* = 4) [38, 45, 46, 51], German Democratic Republic (*n* = 2) [43, 44]), Denmark (*n* = 3) [8, 17, 36], United Kingdom (*n* = 3) [24–26], Finland (*n* = 2) [27, 35], Czech Republic (*n* = 1) [42], Netherlands (*n* = 1) [39], Russia (*n* = 1) [37], Slovenia (*n* = 1) [41] and Sweden (*n* = 1) [47]. The year of study publication ranged from 1962 to 2020. Sixteen studies were published in English, four in German and one each in Dutch and Czech. Detailed information on study characteristics are shown in Additional file 4.

Most studies (*n* = 14) involved only male participants, four studies [24, 35, 52, 53] recruited both sexes and one study [46] included only females. Three studies did not provide any information about the sex of participants, but we presumed that they were predominantly male, as in two studies which investigated professional football players there was no professional women’s league at time of recruitment [39, 41], or due to the nature of the described occupation (manual welders in ship-building) [43]. The studies included professional football players (*n* = 6) [37–39, 41, 46, 47], professional basketball players (*n* = 2) [40, 50], floor layers (*n* = 3) [8, 17, 27], miners (*n* = 2) [26, 42] and one each focused on baggage handlers [36], pipefitters [44], shunters [45], manual welders [43], and farmers [53]. Four studies [24, 25, 35, 51] included workers from different industries or did not target a specific occupational group but reported on knee-straining occupational activities. The comparison groups comprised predominantly persons from the general population or less-exposed workers, e.g. graphic designers or house painters. Six studies [37, 40, 41, 43, 46, 50] did not include a comparison group and only provided prevalence data on meniscal lesions within a specific occupational group.

Most studies provided information on knee-straining exposure based on job description. Only three case-control studies [24, 25, 52] and one cross-sectional study [53] analyzed the risk of specific occupational activities, e.g. kneeling, squatting or lifting and carrying heavy weights, on the development of meniscal lesions. One cohort study [35] divided occupational exposure in light, moderate and heavy work, but the exposure definition included a wide range of not only knee-straining activities. In eleven studies [8, 17, 24–26, 35, 39, 41, 46, 51, 53] the exposure was assessed through questionnaire or interview, whereas only five studies used objective methods, e.g. systematic observations [45], video recording [27] or gaining information from employer registers [36, 37, 43]. Six studies [38, 40, 42, 43, 47, 50] did not report any specific exposure measurement.

Meniscal lesions were predominantly assessed objectively through MRI (*n* = 7) [8, 37, 38, 40, 46, 50, 53], surgically or arthroscopically (*n* = 4) [24, 25, 42, 51] or based on records from hospital register or health insurance data (*n* = 5) [26, 35, 36, 45, 47]. Three studies collected information on meniscal lesions through self-report via standardized interview or questionnaire [27, 39, 41], three studies identified meniscal lesions through clinical examination [17, 43, 44].

### Risk of bias

Only one study [36] had an overall low risk of bias (Table 1). The chronology was judged as high risk of bias in all cases but one, as it was not established due to cross-sectional study design or due to unknown meniscal status of participants at baseline in cohort and case-control studies. Only five studies [27, 36, 37, 44, 45] were rated as having a low risk of bias for exposure definition and measurement, because most often exposure was assessed subjectively (*n* = 11) or detailed information on exposure was lacking. Studies that presented incomplete analyses (*n* = 1) [39] or that only provided prevalence data and did not calculate prevalence ratios (*n* = 13), including seven studies without comparison group, were judged as having a high risk of bias in analysis methods. Most studies (*n* = 17) did not report on any criteria of the minor domains. Conflict of interest was the most common item of all domains rated as “unclear”.

**Table 1:** Risk of bias of the included studies

Study	Major domains^**a**^						Minor domains^**b**^			Overall risk of bias
	1	2	3	4	5	6	7	8	9	
*Cohort studies*										
Kontio et al., 2017 [35]	+	–	+	+	+	–	+	+	+	–
Mikkelsen et al., 2016 [36]	+	+	+	+	+	+	+	+	+	+
*Cross-sectional studies*										
Bezuglov et al., 2019 [37]	+	+	+	–	–	–	+	?	+	–
Behzadi et al., 2017 [38]	–	–	+	+	–	–	?	+	+	–
Brouwer et al., 1981 [39]	–	–	–	–	–	–	+	?	?	–
Hong et al., 2020 [53]	+	–	+	+	+	–	?	+	+	–
Kaplan et al., 2005 [40]	–	–	+	–	–	–	?	?	?	–
Kivimäki et al., 1992 [27]	+	+	–	+	–	–	+	+	?	–
Krajnc et al., 2010 [41]	+	–	–	–	–	–	+	?	+	–
Musialek & Kostal, 1995 [42]	+	–	+	–	–	–	+	?	?	–
Nauwald, 1980 [43]	+	–	–	–	–	–	–	+	?	–
Nauwald, 1986 [44]	+	+	–	–	–	–	–	+	?	–
Pressel, 1982 [45]	+	+	–	–	–	–	+	?	?	–
Prien et al., 2019 [46]	–	–	–	+	–	–	+	+	+	–
Roos et al., 1994 [47]	–	–	+	–	–	–	+	+	?	–
Rytter et al., 2008 [17]	–	–	–	+	+	–	+	+	+	–
Rytter et al., 2009 [8]; Jensen et al., 2012 [49]; Jensen et al., 2012 [48]	–	–	+	+	+	–	–	+	+	–
Walczak et al. (2008) [50]	–	–	+	–	–	–	?	?	?	–
*Case-control studies*										
Baker et al., 2002 [24]	+	–	+	–	+	–	?	+	?	–
Baker et al., 2003 [25]	+	–	–	+	+	–	?	+	?	–
Gotthardt et al., 1995 [51]; Gotthardt, 1997 [52]	–	–	+	+	+	–	?	?	?	–
Sharrard & Liddell, 1962 [26]	–	–	+	+	–	–	+	?	?	–

^a^ Risk of bias due to: (1) Recruitment procedure & follow-up (in cohort studies), (2) Exposure definition and measurement, (3) Outcome source and validation, (4) Confounding and effect modification, (5) Analysis method: methods to reduce research specific bias, (6) Chronology

^b^ Risk of bias due to: (7) Blinding of assessors, (8) Funding, (9) Conflict of interest

+ = low risk; − = high risk;? = unclear

### Prevalence of meniscal lesions

In total, we identified 18 studies that reported the prevalence or incidence rate of meniscal lesions in specific occupational groups exposed to knee-straining activities.

Three studies focused on floor and carpet layers who predominantly worked in kneeling postures on the floor. Kivimäki et al. [27] observed meniscal lesions in 10.1% of participants. Rytter et al. [17] identified meniscal lesions through clinical examination and reported a prevalence rate of 23.9% using the McMurray test and 31.3% palpating the tibiofemoral joint line. In contrast, Rytter et al. [8] identified a much higher rate of meniscal lesions (67.4% medial meniscus lesions, 13.0% lateral meniscus lesions) in the same population of floor layers using MRI. In one study each, the rate of meniscal lesions was examined in other occupational groups which were also mainly exposed to kneeling activities: miners (25.6 per 10,000 person years) [42], manual welders (right knee: 19%; left knee: 17%) [43] and pipe-fitters (right knee 11.8%; left knee: 6.9%) [44]. One study investigated the prevalence of meniscal lesions in shunters who were exposed to various knee-straining activities, e.g. squatting, walking on slippery and uneven surfaces or running and jumping [45]. Data on meniscal lesions were collected based on health insurance medical records, but only 0.5% suspicious meniscal lesions and 0.4% possible meniscal lesions were identified. In Korean farmers who mainly worked in a squatting position and lifting heavy weights, Hong et al. [53] found meniscal tears in 54.5% by MRI. Investigating heavy lifting in a kneeling or squatting position as a risk factor for meniscal lesions, Mikkelsen et al. [36] described an incidence rate of 62.16 per 10,000 person years in airport baggage handlers. Kontio et al. [35] did not describe a specific occupation but reported meniscal lesions in 5.1% of individuals with light, 5.9% in those with moderate and 3.4% in those with heavy physical work.

Only three studies that investigated workers exposed to repetitive knee-straining activities reported the localisation of meniscal lesions. According to Musialek et al. [42] 84.6% of identified meniscal lesions in miners occurred at the medial meniscus and 15.4% in the lateral meniscus. Rytter et al. [8] reported a rate of 83.8% medial and 16.2% lateral meniscal lesions in floor layers. A higher rate of medial meniscal lesions was also observed in farmers (female: 54.0% medial, 25.0% lateral; male: 44.1% medial, 13.9% lateral) [53]. These findings indicated that in workers predominately exposed to kneeling or squatting activities, lesions in the medial meniscus were more common than in the lateral meniscus.

Eight included studies presented information on meniscal lesions among former elite team sports athletes. Six studies [37–39, 41, 46, 47] focused on former professional football players, and reported a prevalence ranging from 16.9 to 69.4%. The findings from Bezuglov et al. [37], Brouwer et al. [39] and Prien et al. [46] indicated that in professional football players both, the medial and the lateral meniscus were affected equally. Two studies investigated the occurrence of meniscal lesions in former professional basketball players. Walczak et al. [50] found asymptomatic meniscal changes in 53.6% of the athletes. Kaplan et al. [40] reported meniscal injuries in 20% of examined knees, noting that in professional basketball players the medial meniscus was affected most frequently.

The complete data extraction of included studies is presented in Additional file 4 and Additional file 5.

### Work-related risk factors

The statistical analyses combined the results of nine studies. Due to a missing control group, six cross-sectional studies were not eligible for meta-analysis [37, 40, 41, 43, 46, 50]. Further, we excluded the results from Rytter et al. [17] as they investigated the same population as Rytter et al. [8] by using clinical examination for outcome measurement. We did not calculate prevalence ratios for the studies from Behzadi et al. [38] because of missing information according the response in controls, and Brouwer et al. [39] as they did not report the prevalence of meniscal lesions in the control group. The results from Kontio et al. [35], Pressel [45] and Hong et al. [53] were excluded because exposure was not comparable to other studies. Although Gotthardt et al. [51] reported on specific occupational activities, e.g. kneeling or squatting, information on duration, intensity and frequency was lacking.

**Fig. 2:**
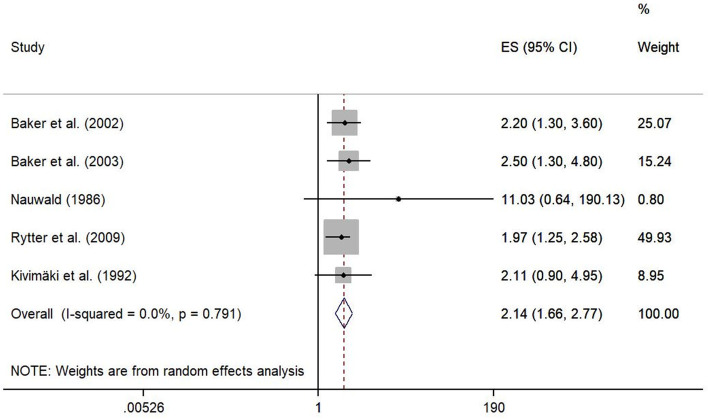
Forest plot of studies regarding the risk of kneeling and the development of meniscal lesions

**Fig. 3:**
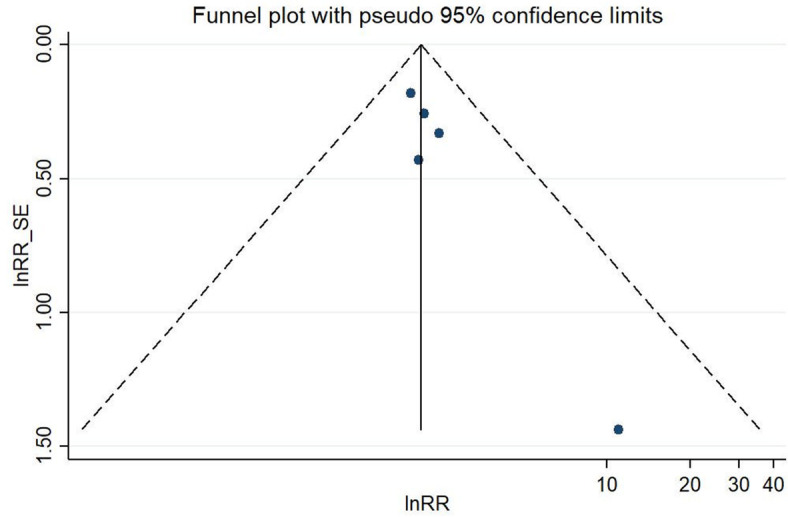
Funnel plot of effect estimates included in the meta-analysis “risk of kneeling” (Fig. 2)

A meta-analysis from five studies indicated that occupational kneeling is associated with the development of meniscal lesions (ES 2.14, 95% CI 1.66–2.77). Baker et al. [24] and Baker et al. [25] compared kneeling > 1 h per day to kneeling less than 1 h per day for at least 12 months up to the onset of symptoms, whereas the other three studies defined exposure through job title. Both, working as a floor layer [8, 27] as well as working as a pipe fitter [44] involved mainly activities in kneeling positions. The exposure groups were compared to persons without any knee-straining activities or defined occupational groups (graphic designers and house painters) whose work did not include any knee demands. With a mean duration of employment of 25 years in pipe fitters [44] as well as 29.6 years [8] and 14.7 years [27] in floor layers, the exposure duration was high. Heterogeneity across study results was considered unimportant (I^2^ = 0.0%, *p* = 0.791) (Fig. 2). The funnel plot presented in Fig. 3 was approximately symmetrical, but Egger’s test was significant (*p* = 0.04). Bias seemed to primarily occur due to the study from Nauwald [44], which had wide confidence intervals as there was no meniscal lesion present in the comparison group.

**Fig. 4:**
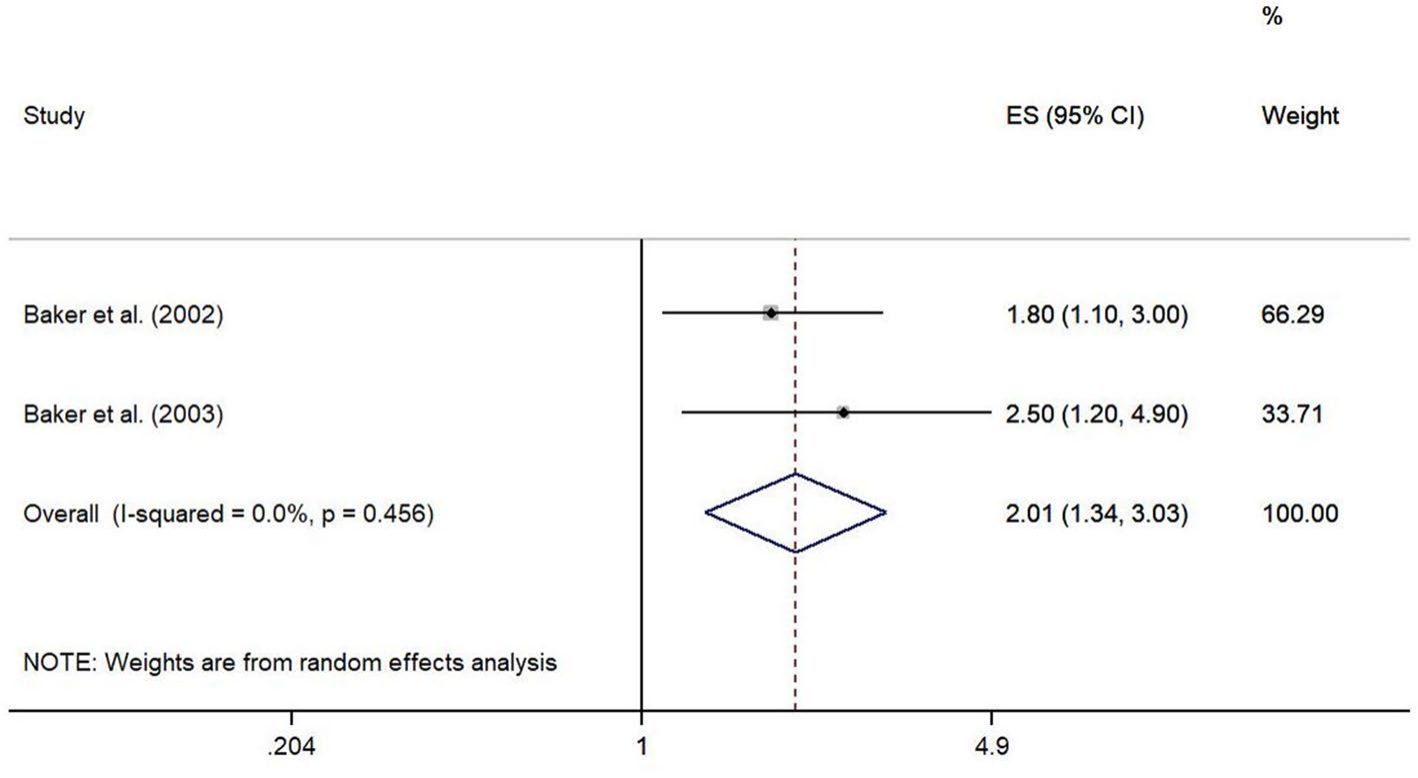
Forest plot of studies regarding the risk of squatting > 1 h per day and the development of meniscal lesions

Based on the results of two case-control studies [24, 25], occupational squatting > 1 h per day compared to squatting less than 1 h per day for at least 12 months up to the onset of symptoms showed a significant association with the development of meniscal lesions (ES 2.01, 95% CI 1.34–3.03). No heterogeneity was observed (I^2^ = 0.0%, *p* = 0.456) (Fig. 4).

**Fig. 5:**
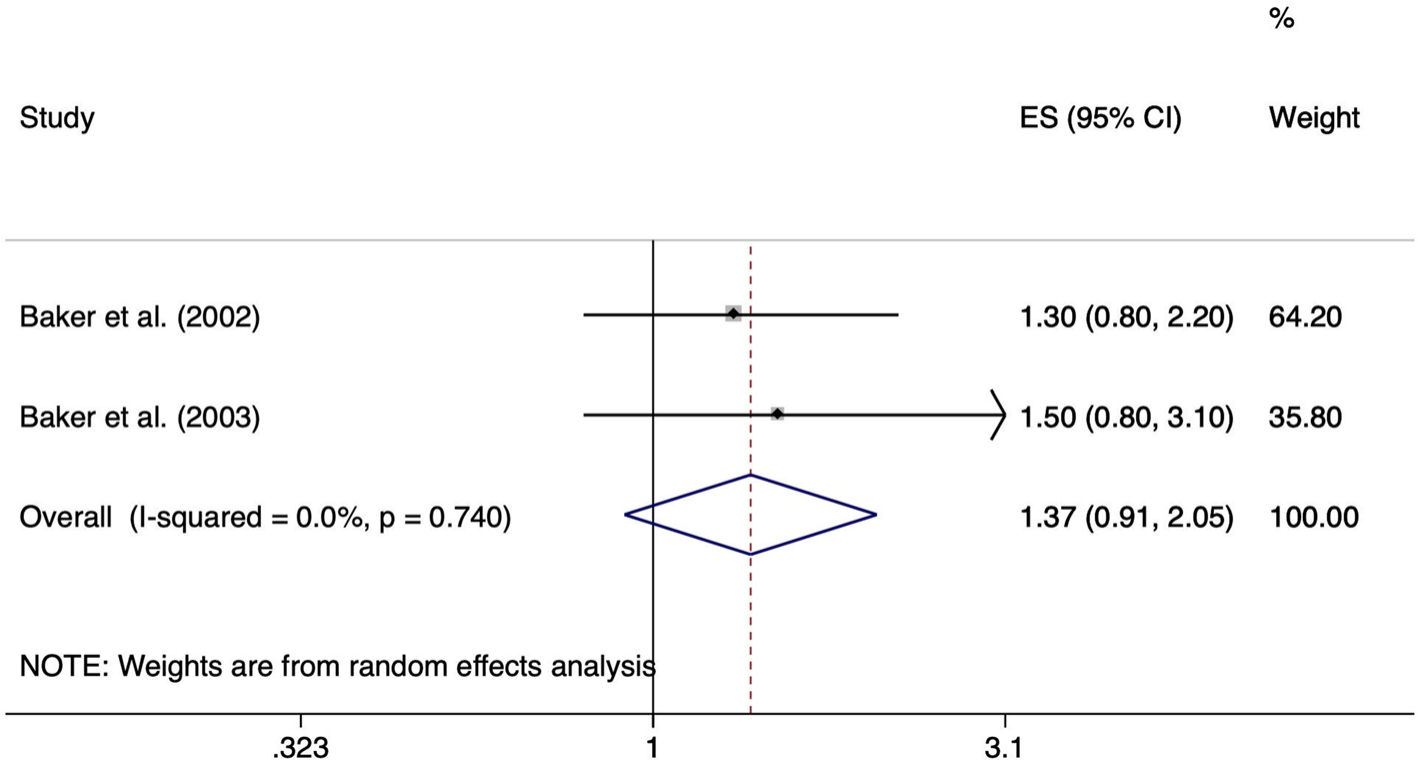
Forest plot of studies regarding the risk of standing or walking > 2 h per day and the development of meniscal lesions

The pooled ES over two studies [24, 25] for individuals standing or walking > 2 h per day at work compared to those standing or walking less than 2 h per day for at least 12 months up to the onset of symptoms was not statistically significant (ES 1.37; 95% CI 0.91–2.05). Heterogeneity across study results was considered unimportant (I^2^ = 0.0%, *p* = 0.740) (Fig. 5).

**Fig. 6:**
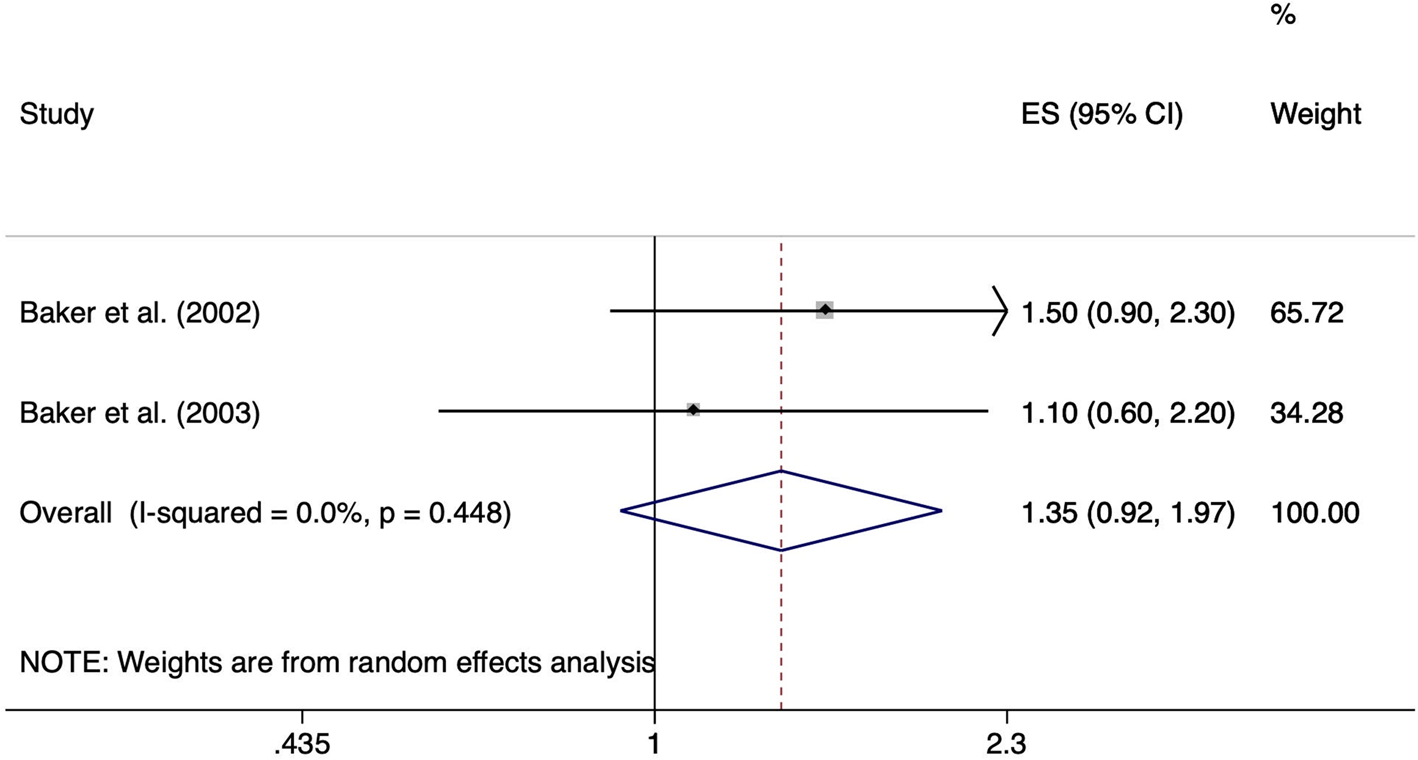
Forest plot of studies regarding the risk of walking > 2 miles per day and the development of meniscal lesions

The risk of walking > 2 miles per day compared to walking less than 2 miles per day for at least 12 months to the upset of symptoms was reported in two case-control studies [24, 25]. Meta-analysis provided a non-significant ES of 1.35 (95% CI 0.92–1.97). Heterogeneity was considered unimportant (I^2^ = 0.0%, *p* = 0.448) (Fig. 6).

**Fig. 7:**
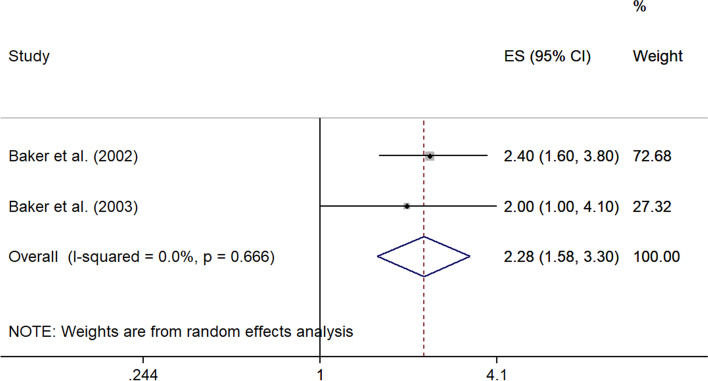
Forest plot of studies regarding the risk of climbing > 30 flights of stairs per day and the development of meniscal lesions

A meta-analysis of two studies [24, 25] that assessed the risk of climbing > 30 flights of stairs per day versus climbing < 30 flights of stairs per day resulted in an ES of 2.28 (95% CI 1.58–3.30), indicating a significant association with the development of meniscal lesions. No heterogeneity across study results was observed (I^2^ = 0.0%, *p* = 0.666) (Fig. 7).

**Fig. 8:**
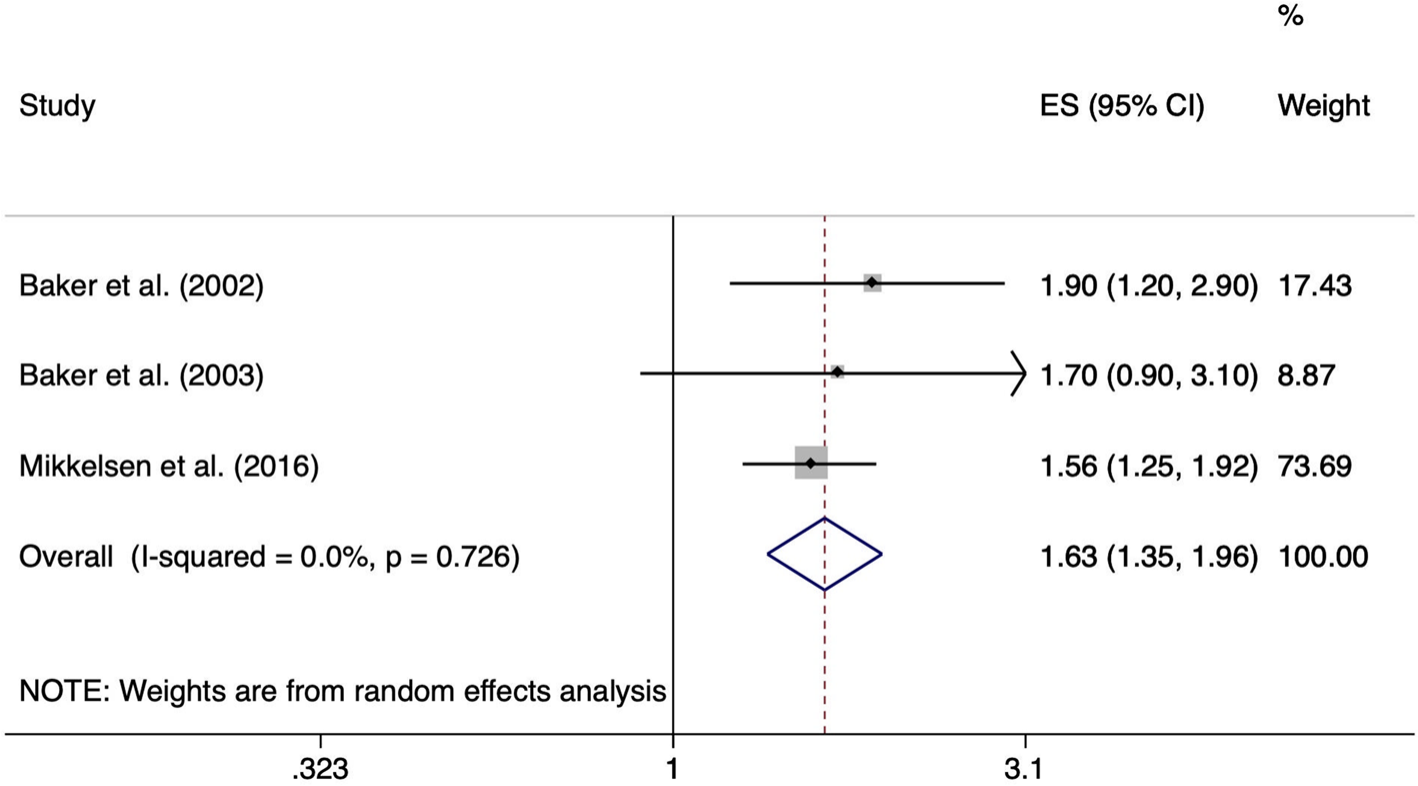
Forest plot of studies regarding the risk of lifting and carrying weights ≥10 kg and the development of meniscal lesions

**Fig. 9:**
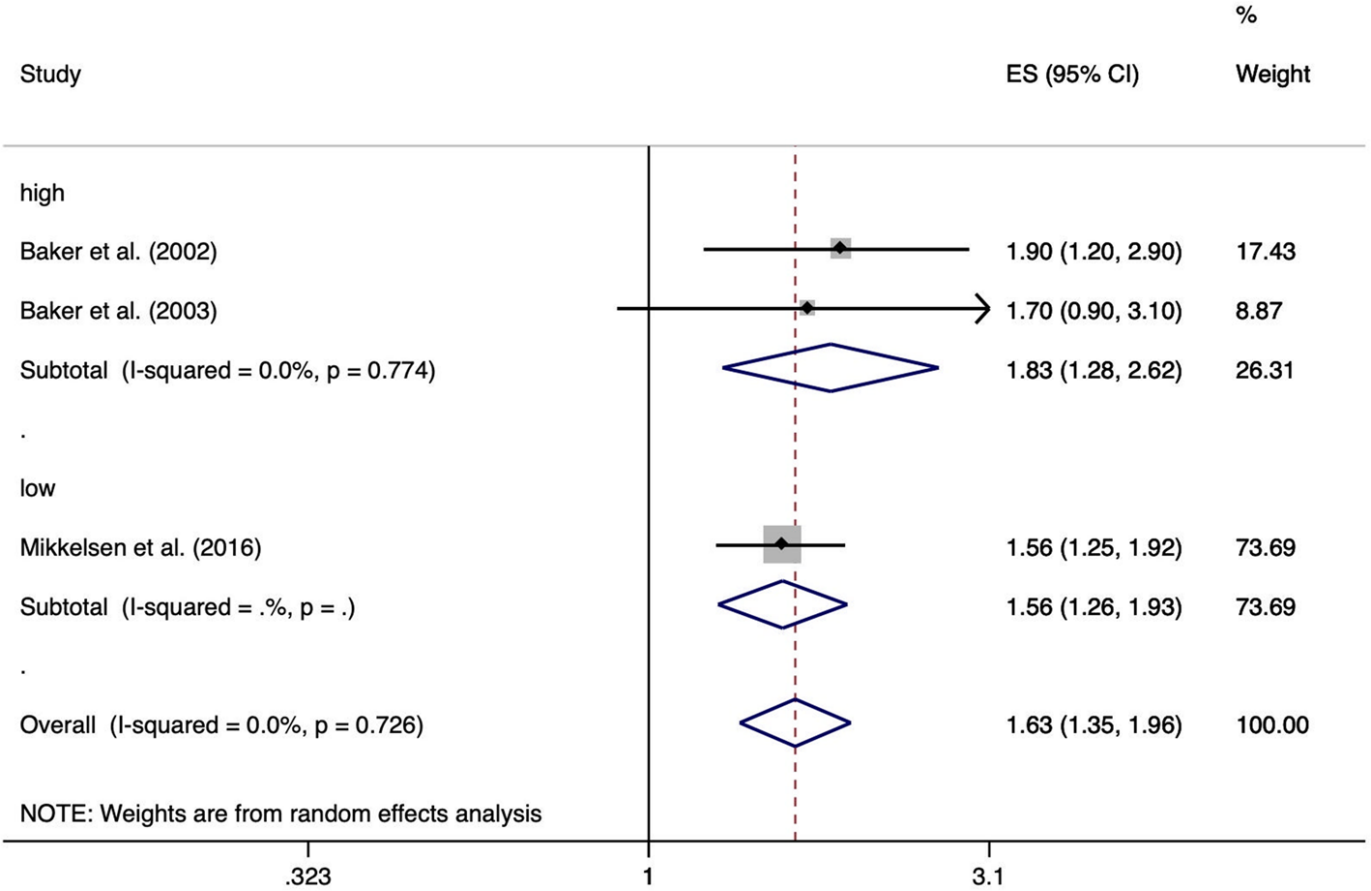
Forest plot of studies regarding the risk of lifting and carrying weights ≥ 10 kg and the development of meniscal lesions by high and low risk of bias

**Fig. 10:**
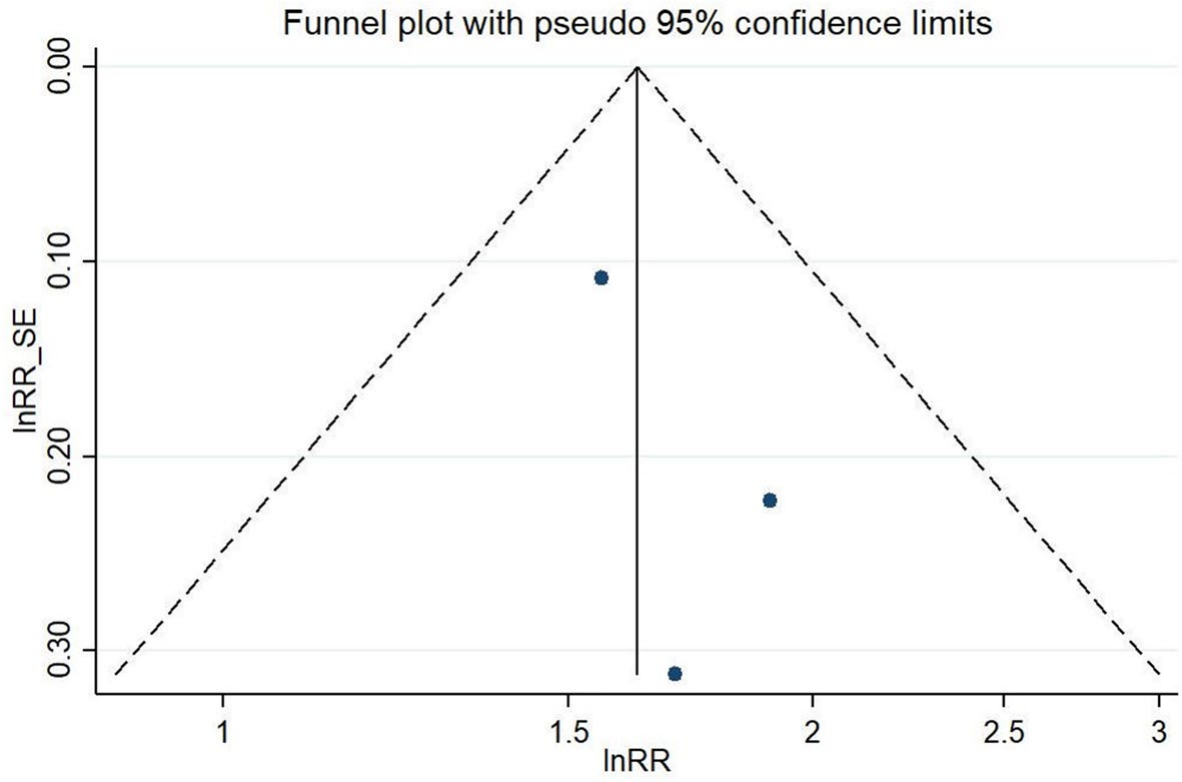
Funnel plot of effect estimates included in the meta-analysis “risk of lifting and carrying weights ≥ 10 kg” (Fig. 8)

Three of the included studies reported on the risk of lifting or carrying weights ≥ 10 kg. Baker et al. [24] and Baker et al. [25] compared individuals lifting or carrying weights ≥ 10 kg more than 10 times per week with those lifting or carrying weights ≥ 10 kg less than 10 times per week for at least 12 months up to the onset of symptoms. Mikkelsen et al. [36] compared baggage handlers, who had to load or unload baggage pieces with an average weight of 15 kg from baggage carts and baggage containers, with non-baggage handlers. The analysis resulted in a significant ES of 1.63 (95% CI 1.35–1.96). We observed no important heterogeneity across study results (I^2^ = 0.0%, *p* = 0.726) (Fig. 8). Figure 9 shows the risk of lifting and carrying weights ≥ 10 kg separated according studies’ risk of bias. Funnel plot analysis and Egger’s test (*p* = 0.44) for asymmetry suggested absence of publication bias (Fig. 10).

**Fig. 11:**
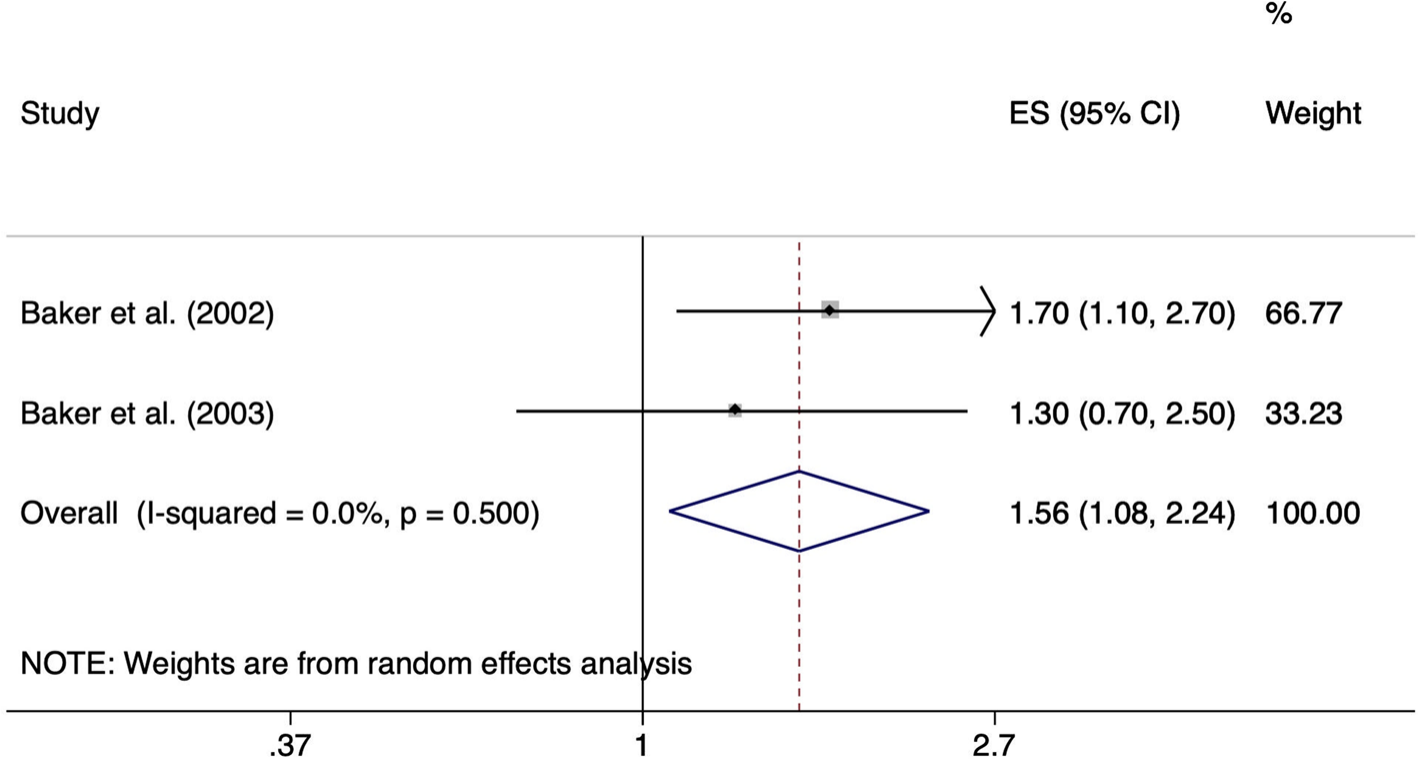
Forest plot of studies regarding the risk of lifting and carrying weights ≥ 25 kg and the development of meniscal lesions

Based on the studies from Baker et al. [24] and Baker et al. [25], for lifting or carrying weights ≥ 25 kg more than 10 times per week compared to lifting or carrying weights ≥ 25 kg less than 10 times per week for at least 12 months up to the onset of symptoms, there is a positive association with the development of meniscal lesions (ES 1.56, 95% 1.08–2.24). Heterogeneity across study results was considered unimportant (I^2^ = 0.0%, *p* = 0.500) (Fig. 11).

**Fig. 12:**
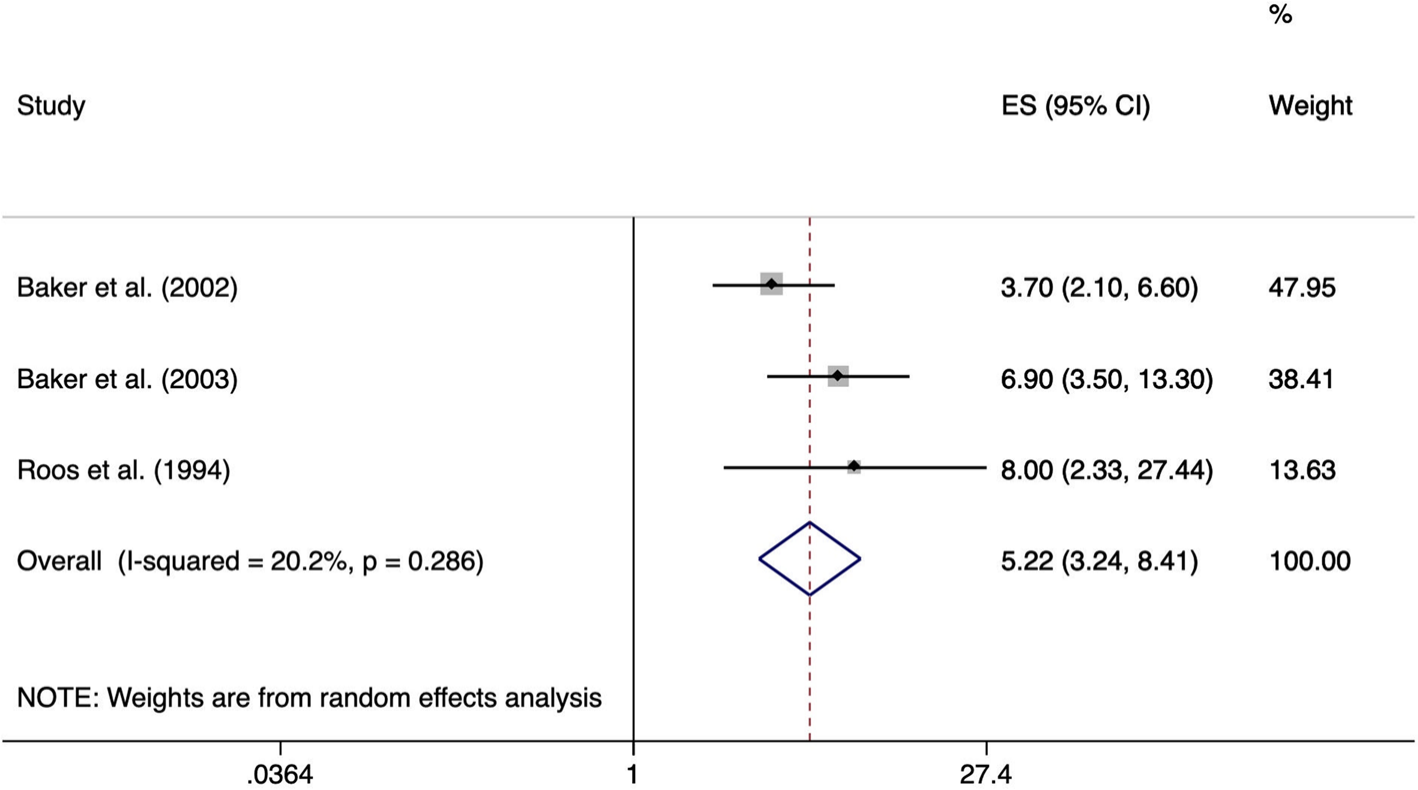
Forest plot of studies regarding the risk of playing football and the development of meniscal lesions

**Fig. 13:**
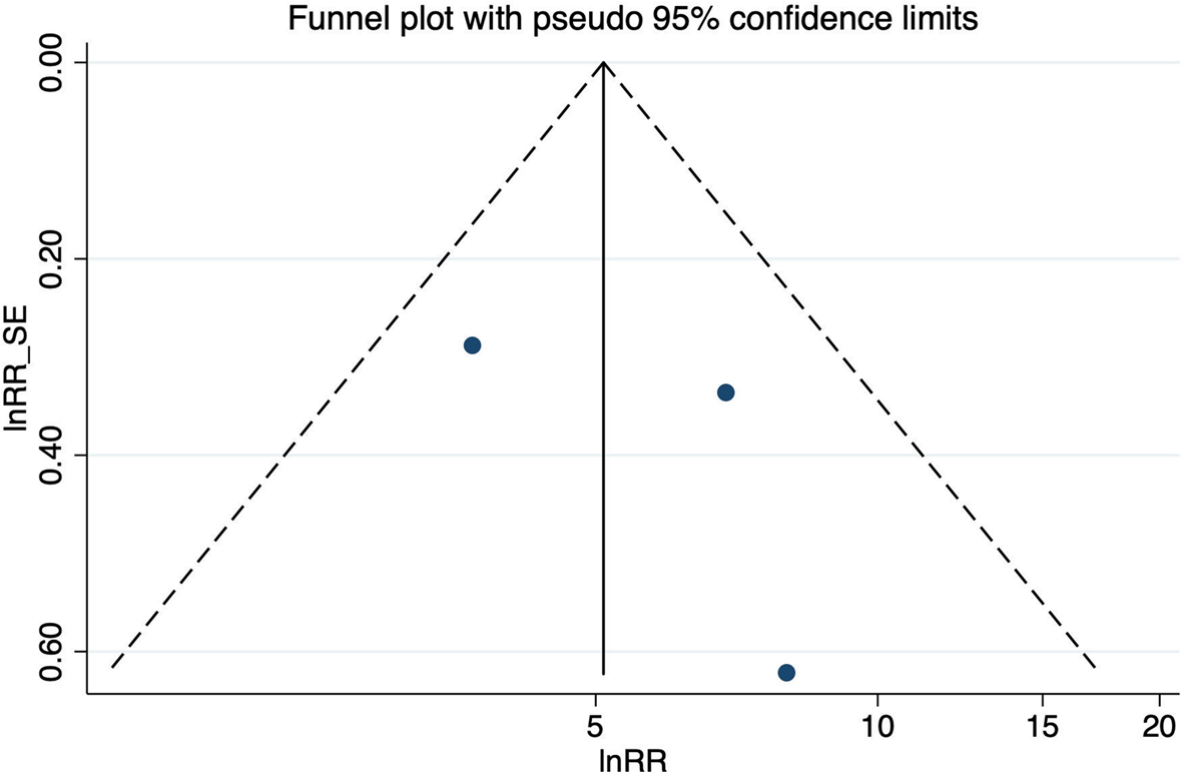
Funnel plot of effect estimates included in the meta-analysis “risk of playing football” (Fig. 12)

Three studies evaluated the association between playing football and the development of meniscal lesions. In the study from Roos et al. [47] former professional football players who had played at least until age 25 were compared to age-matched controls whose former football activity was unknown. Baker et al. [24] and Baker et al. [25] compared playing football (at least five times) in the 12 months leading up to the onset of their symptoms to individuals not playing football. Thus, presumably not only football players on professional level were included. The exposure duration in years was not reported in any study. Given that football players started their professional career with the age of 19 [54], the mean exposure duration in the study of Roos et al. [47] is about 6 years. The meta-analysis yielded a statistically significant ES of 5.22 (95% CI 3.24–8.41). Heterogeneity was considered unimportant (I^2^ = 20.2%, *p* = 0.286) (Fig. 12). No evidence of publication bias was given by funnel plot and Egger’s test (*p* = 0.54) (Fig. 13).

**Fig. 14:**
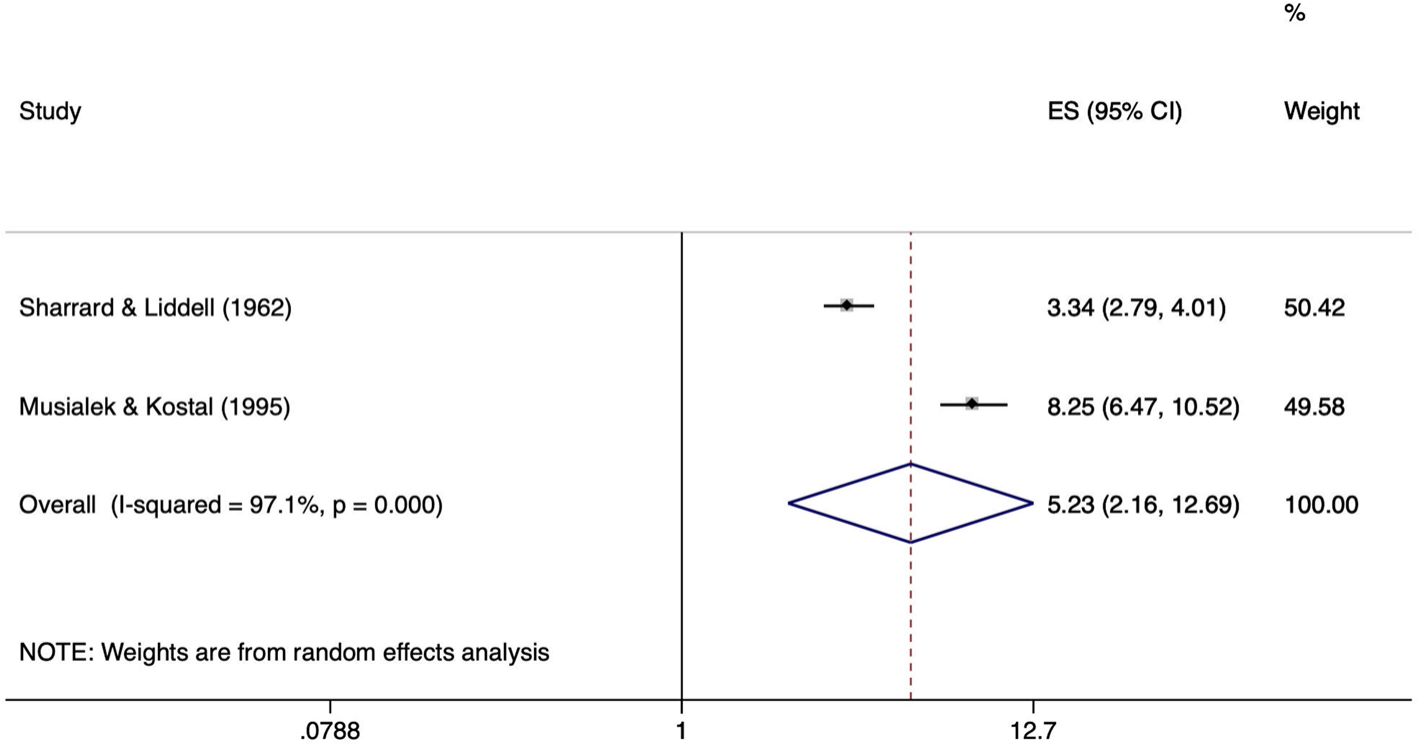
Forest plot of studies regarding the risk of mining and the development of meniscal lesions

Two studies [26, 42] compared individuals working as a hard coal miner with a group of non-miners. There was no information on exposure duration. In one study [42] the mean age of the miners with meniscal lesions was 34.1 years. Given that miners started working after finishing school at the age of 16–18 years, this would correspond to a mean exposure time of about 16–18 years. A statistically significant pooled ES of 5.23 (95% CI 2.16–12.69) indicated an increased risk of meniscal lesions in miners, but statistical heterogeneity was considered substantial (I^2^ = 97.1%, *p* < 0.001) (Fig. 14).

**Fig. 15:**
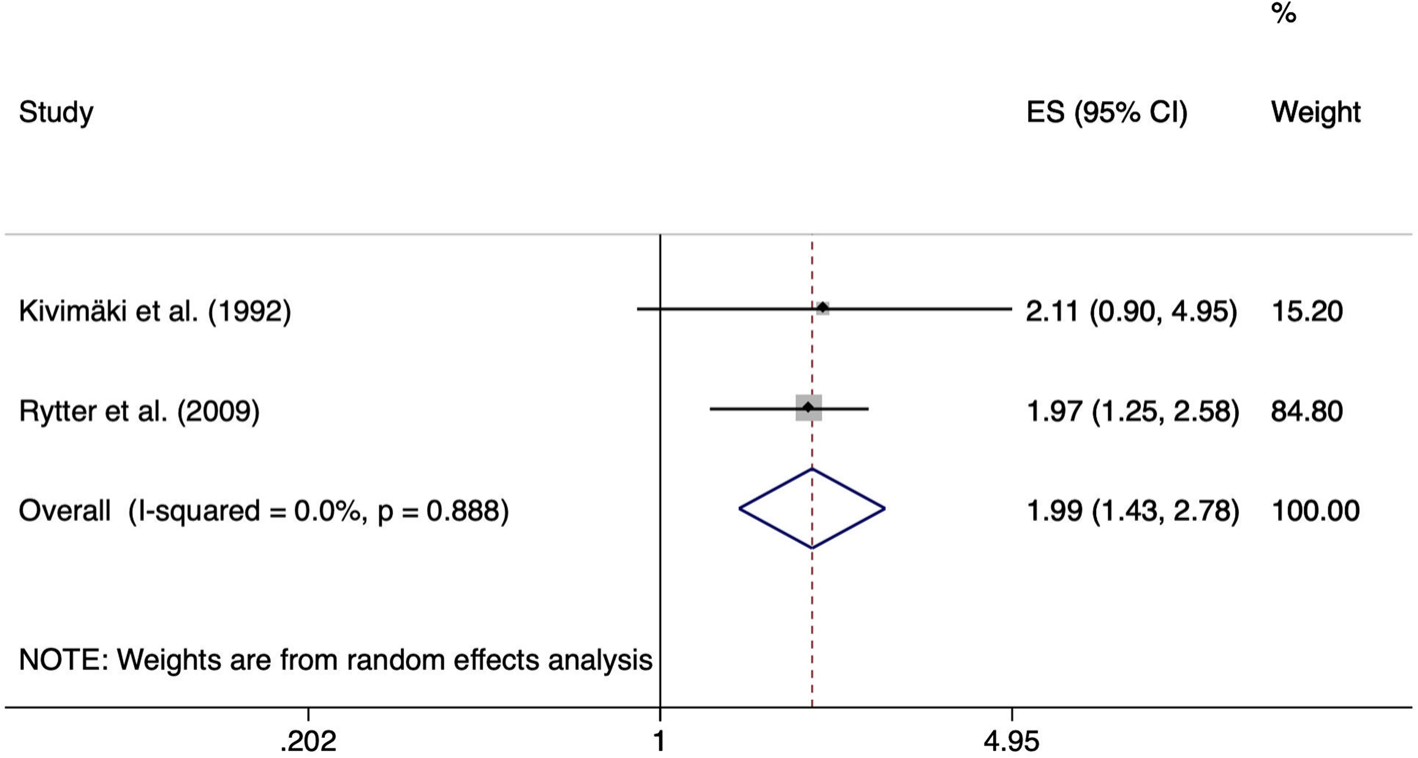
Forest plot of studies regarding the risk of working as a floor layer and the development of meniscal lesions

Working as a floor layer was positively associated with the development of meniscal lesions with a pooled ES of 1.99 (95% CI 1.43–2.78). With an average working time of 14.7 years [27] and 29.6 years [8], respectively, the duration of exposure was high. Floor layers were compared to a group of house painters or graphic designers, who were not exposed to knee-straining activities. Heterogeneity across study results was considered unimportant (I^2^ = 0.0%, *p* = 0.888) (Fig. 15).

#### Other risk factors

Only one study [25] compared individuals lifting or carrying weights ≥ 50 kg more than 10 times per week to those lifting or carrying weights ≥ 50 kg less than 10 times per week for at least 12 months up to the onset of symptoms and found a significant association with the development of meniscal lesions (OR 2.4; 95% CI 1.4–4.2). Further, Gotthardt et al. [51] investigated working on ladders (OR 0.97; 95% CI 0.54–1.74), shock-like exposure (OR 1.11; 95% CI 0.81–1.52) and walking on uneven surfaces (OR 1.13; 95% CI 0.84–1.52) as possible risk factors for meniscal lesions. However, there was no information on duration, intensity and frequency of exposure and no significant associations were found.

A dose-response analysis was not possible for any exposure due to insufficient data.

### Quality of evidence assessment

The overall quality of evidence according to GRADE varied between low for standing or walking, lifting or carrying weights ≥ 25 kg and working as a floor layer, and moderate for kneeling, squatting, climbing stairs, lifting and carrying weights ≥ 10 kg, playing football and working as a hard coal miner (Table 2). All but one investigated risk factors were downsized for one level because the analyses included studies of low methodological quality. We did not downgrade one level for quality of study limitations for the risk of lifting and carrying weights ≥ 10 kg because one low risk of bias study showing statistically significant results was included. For six risk factors we upgraded one level for ES as the effect estimate was larger than 2.0 and even twice for the risk of playing football and working as a hard coal miner as the ES was larger than 5.0.

**Table 2:** Assessment of evidence for the risk of studied outcomes based on Grades of Recommendations, Assessment, Development and Evaluation framework (GRADE)

Risk	Quality of study limitations: ↓	Indirectness of evidence: ↓	Inconsistency: ↓	Imprecision range confidence interval effect size > 2.0: ↓	Publication bias yes: ↓	Effect estimate > 2.0: ↑ > 5.0: ↑↑	Dose-response effect: ↑	Residual confounding: ↑	Overall certainty (high, moderate, low)
Kneeling	yes ↓^a^	no (−)	no (−)	no (−) 1.66–2.77	no (−)^b^	yes ↑ 2.14	no (−)	no (−)	moderate
Squatting > 1 h per day	yes ↓^a^	no (−)	no (−)	no (−) 1.34–3.03	no (−)	yes ↑ 2.01	no (−)	no (−)	moderate
Standing or walking > 2 h per day	yes ↓^a^	no (−)	no (−)	no (−) 0.91–2.05	no (−)	no (−) 1.37	no (−)	no (−)	low
Walking > 2 miles per day	yes ↓^a^	no (−)	no (−)	no (−) 0.92–1.97	no (−)	no (−) 1.35	no (−)	no (−)	low
Risk of climbing > 30 flights of stairs per day	yes ↓^a^	no (−)	no (−)	no (−) 1.58–3.30	no (−)	yes ↑ 2.28	no (−)	no (−)	moderate
Risk of lifting and carrying ≥ 10 kg	no (−)^c^	no (−)	no (−)	no (−) 1.35–1.96	no (−)	no (−) 1.63	no (−)	no (−)	moderate
Risk of lifting and carrying ≥ 25 kg	yes ↓^a^	no (−)	no (−)	no (−) 1.08–2.24	no (−)	no (−) 1.56	no (−)	no (−)	low
Risk of playing football	yes ↓^a^	no (−)	no (−)	yes ↓ 3.24–8.41	no (−)	yes ↑↑ 5.24	no (−)	no (−)	moderate
Risk of mining	yes ↓^a^	no (−)	no (−)	yes ↓ 2.16–12.69	no (−)	yes ↑↑ 5.23	no (−)	no (−)	moderate
Risk of floor layers	yes ↓^a^	no (−)	no (−)	no (−) 1.43–2.78	no (−)	no (−) 1.99	no (−)	no (−)	low

^a^ All studies had a high risk of bias

^b^ Egger’s test *p* = 0.04. However, Nauwald et al. 1986 is the reason due to its wide confidence intervals due to the zero for the comparison group

^c^ 2/3 studies had a high risk of bias, and high risk of bias studied increased the RR (High risk RR = 1.83; 95% CI 1.28–2.62; Low risk RR = 1.56; 95% CI 1.26–1.93), but: low risk of bias studies was statistically significant

## Discussion

This systematic review evaluated the possible relationship between occupational knee-straining exposures and the development of meniscal lesions. Twenty-two studies met our inclusion criteria of which nine studies were eligible for meta-analysis. Significant associations between occupational risk factors and the development of meniscal lesions were found for kneeling, squatting, climbing stairs, lifting and carrying weights ≥ 10 kg, lifting and carrying weights ≥ 25 kg and specific occupational groups (professional football players, miners and floor layers). The overall quality of evidence according GRADE was moderate for kneeling, squatting, climbing stairs, lifting and carrying weights ≥ 10 kg, playing football and working as a hard coal miner, and low for standing or walking, lifting or carrying weights ≥ 25 kg and working as a floor layer.

The findings of our review are in line with a previous review by Snoeker et al. [14] that also identified kneeling and squatting, climbing stairs, lifting and carrying weights and playing football to be associated with meniscal lesions. But in contrast, we did not find a statistically significant association in walking > 2 miles per day and standing or walking > 2 h per day, as we used the adjusted risk estimates to reduce bias due to confounding. Reid et al. [55] concluded that squatting should be considered an occupational risk factor, which is consistent with our findings. Based on a meta-analysis including two studies [26, 42], we found moderate evidence that working as a hard coal miner is associated with the development of meniscal lesions, as previously suggested by Mc Millan and Nichols [56].

Due to insufficient data, we did not conduct a dose-response analysis. However, we investigated the association between lifting and carrying weights and the development of meniscal lesions, but no considerable differences were found between individuals exposed to weights ≥ 10 kg (ES 1.63, 95% CI 1.35–1.96) and ≥ 25 kg (ES 1.56, 95% 1.08–2.24). But since exposure is defined not only by intensity, future studies should focus on frequency and duration of lifting and carrying weights. In both case-control studies [24, 25] the exposure frequency was kept very low with lifting or carrying weights at least ten times a week and information on exposure duration was lacking.

We identified three studies that investigated the localisation of structural changes in former elite footballers’ knee joints, reporting a high prevalence rate of meniscal lesions. In workers exposed to kneeling activities the medial meniscus is more frequently affected, whereas in professional football players meniscal lesions occurred equally in the lateral and medial meniscus. A possible explanation could be the different forces at the knee joint that result from different exposures. While workers exposed to kneeling or squatting activities, e.g. miners or floor layers, use to work in an awkward position that lead to high biomechanical stresses on the joint structures, athletes in high-speed contact sport as football, basketball or handball are exposed to highest intensity of joint impact with twisting and torsional loading. However, professional basketball players did not show the same prevalence of meniscal lesions in lateral meniscus as professional football players, but findings were based on only one study.

We performed an extensive literature review using a comprehensive search string in three databases and even included unpublished studies (grey literature). There were no language or time restrictions to ensure the inclusion of as many relevant studies as possible. A strength of our research methods was that the appraisal of titles, abstracts and full texts, data extraction and the assessment of study quality were carried out independently by two researchers. We used strict selection criteria and studies with no information on the response were excluded due to the potential for selection bias. However, there was a large heterogeneity among the included studies and only nine studies were eligible for meta-analysis, with a low number of studies within each exposure category. Although our results provide evidence of an association between occupational risk factors and the development of meniscal lesions, some limitations of our meta-analysis must be addressed.

The precision of the effect estimators is reduced by the heterogeneity of the exposure definition and measurement in individual studies included in the meta-analysis. The exposure definition varied using either job titles or the description of specific working tasks. We included populations from different occupations and occupational sectors. As even the spectrum of daily exposure within a single job can vary greatly due to different work content, specific characteristics of workplaces and individual preferences of working postures [57], a large heterogeneity in described exposure can be assumed. Moreover, an adequate reporting of exposure duration, frequency and intensity was lacking in most studies. Information on the exposure was predominantly assessed via self-report using questionnaires or interviews. Although this method of measurement is a low cost and easy way to assess especially retrospective exposures of work shifts decades ago, the validity is low. Ditchen et al. [58] stated that self-report showed good to acceptable quality in identifying knee postures but mostly poor to very poor quality in quantifying the load. More objectively methods for exposure assessment are workplace observations or video-recordings as used in the study from Kivimäki et al. [27]. But since only specific working sequences are filmed and the duration of knee-straining postures is extrapolated to an entire work shift, there is a risk of overestimation. The use of task based measurement data in combination with self-reported diary information may be a cost efficient and valid alternative [57]. Another promising approach for long-term technical measurement of occupational knee-straining activities is the use of wireless accelerometers that provide valid information on kneeling and squatting under laboratory conditions, and for kneeling as well under normal working conditions [59].

This systematic review only examined the relationship between occupational physical activities and meniscal lesions; other potential risk factors such as knee-straining leisure-time physical activity were not studied. The possible bias induced by confounding factors of the associations examined was reduced by using the fully adjusted risk estimations of the individual studies. However, only three of the nine studies included in the meta-analyses had adjusted for leisure-time physical activities and the possibility of residual confounding cannot be excluded. It is therefore recommended that future studies assess both occupational and leisure-time activities, so that independent relationships to both can be examined.

Moreover, the overall methodological quality in all but one of the included studies was low. Besides the insufficient reporting of exposure as described above, the chronology was the most important domain limiting study quality. Only Mikkelsen et al. [36] reduced risk of bias from existing meniscal lesions at baseline by excluding participants from the basic cohort with an outcome before first date of employment. There is little research determining the prevalence of meniscal lesions in asymptomatic, unexposed individuals at the beginning of employment. Two studies [60, 61] that investigated the knee joints of adolescent volunteers (average age < 20 years) not exposed to regular sporting activities did not find any meniscal lesion in participants, whereas Jerosch et al. [62] reported grade 2 meniscal lesions in 19.4% of unexposed volunteers under the age of 16, according to the classification of Glashow et al. [63]. However, meniscal lesions that reached the upper or lower articular surface or led to fissuration or fragmentation of the meniscus (grade 3 and 4) were not found. Ludman et al. [64] described Grad 3 meniscal lesions according to the classification of Stoller et al. [65] in 11.5% of the posterior horns of the medial meniscus in 26 knees of unexposed volunteers aged 18–23 years. Although in all studies the number of participants was low and selection bias due to unreported response may have existed, these findings indicated that meniscal lesions can be present in asymptomatic unexposed individuals already at the beginning of employment. Thus, chronology was considered important.

These limitations could have affected the results of our review and limit the generalizability of our findings. To prevent recall bias, future studies should use more objective measurements of exposure and provide detailed information on intensity, duration, and frequency of risk factors.

However, our results indicate an association between occupational activities and the development of meniscal lesions. Prevention of knee disorders at work may be beneficial to reduce sickness absence and work-related health care costs. Until today, there is little research about how to prevent work-related knee disorders. Redistributing mechanical loads and to minimize the time spend in kneeling working positions are important strategies to reduce structural knee damages. Porter et al. [66] indicated that kneepads could decrease the pressure on the bony structures of the knee by distributing the forces across more surface area. However, peak pressures over key anatomic structures of the knee (e.g. bursa sac) have still been reported, and new kneepad designs that redistribute the pressure across a greater surface area are needed. Another approach to prevent discomfort and pain related to occupational squatting is the use of leg support exoskeletons, which reduce worker’s muscle activity around the knee and thus, is expected to reduce the compressive loading at the joint [67]. In the floor-laying trade, different new methods and tools (e.g. electrical screed levelling machines) have been introduced to carry out many of the job tasks from an upright work position and thus, reducing physical demands at work [68, 69]. According to Jensen and Friche [68], workers who had used the new working methods more than 1 year were less likely to report severe knee complaints compared with floor layers who had used the new working methods less than 1 year (knee complaints > 30 days during the previous 12 months: OR 1.49, 95% CI 1.0–2.23; locking of the knee: OR 1.23, 95% CI 0.88–1.71; moderate-to-severe knee pain: OR 1.42, 95% CI 0.93–2.16). But implementation of new methods is difficult and long-lasting, and a participatory approach is recommended.

## Conclusion

In conclusion, we found consistent evidence of an increased risk of meniscal lesions by occupational knee-straining activities (kneeling, squatting, climbing stairs, lifting or carrying heavy weights) as well as specific occupational groups (professional football player, floor layers, and hard coal miners). These results are based on only nine studies, and overall quality of evidence was moderate to low. Nevertheless, the development of strategies that reduce mechanical loads on the knee joints and minimize time spend in knee-straining positions seems important to prevent knee disorders at workplace. Further studies providing consistent information on exposure definition and measurement are recommended to better understand the relationship between occupational risks and meniscal lesions and to allow conclusions on a dose-response relationship.

## Supplementary Information

**Additional file 1.** Search strategy.

**Additional file 2.** Risk of bias schema.

**Additional file 3.** List of excluded studies.

**Additional file 4.** Study characteristics of included studies.

**Additional file 5.** Results shown in included studies.

## Data Availability

Not applicable. The data used for analysis was retrieved from openly published studies listed in our manuscript.

## Abbreviations

ACL: Anterior cruciate ligament
ES: Effect size
MeSH: Medical Subject Heading
MRI: Magnetic Resonance Imaging
MOOSE: Meta-analyses Of Observational Studies in Epidemiology
OR: Odds ratio
PRISMA: Preferred Reporting Items for Systematic Reviews and Meta-Analyses
