# Supplementary material for: Occupational risk factors for meniscal lesions: a systematic review and meta-analysis
Source: BMC Musculoskelet Disord. 2021 Dec 15;22:1042. doi: 10.1186/s12891-021-04900-7 (PMC8672613; doi:10.1186/s12891-021-04900-7)
Supplement: Supplementary file 1 — Additional file 1. Search strategy. [file 12891_2021_4900_MOESM1_ESM.pdf]

Additional file 1: Search strategy

1. Medline (via Ovid): 28.02.2020

#1	Work/ or workplace/ or workload/ or employment/ or occupations/ or occupational diseases/ or occupational exposure/ or occupational medicine/ or (work* or employ* or occupation* or job* or industr*).ti,ab.
#2	Lifting/ or (lifting or kneeling or carrying or climbing or squatting or crawling or stooping or ((running or moving or walking or jumping) adj2 ((hard or uneven or rough or bumpy or slippery) adj2 (surface* or ground* or terrain*))) or (knee adj (buckling or shifting or slipping)) or (shear* adj (stress* or force* or motion*)) or pivoting or twisting).ti,ab.
#3	Mining/ or coal mining/ or gardening/ or agriculture/ or (mining or construction or tiling or shunting or paving or roofing or gardening or farming).ti,ab.
#4	Miners/ or farmers/ or (miner* or farmer* or ((blue-collar or factory or coal-face or construction or road or concrete or shipyard or metal or asphalt) adj (worker* or laborer* or labourer*)) or ((floor or carpet or parquet or pipe) adj (layer* or fitter*)) or pipefitter* or woodworker* or tiler* or tile setter* or ((stove or oven) adj (maker* or builder* or fitter* or manufacturer*)) or shunter* or screed installer* or baggage handler* or bricklayer* or mason* or builder* or paver* or roofer* or slater* or painter* or decorator* or ((electrical or television or computer) adj engineer*) or cleaner* or laborer* or labourer* or childcare or joiner* or carpenter* or motor mechanic* or maintenance fitter* or installer* or welder* or shipbuilder* or docker* or gardener* or millwright* or fitter* or boilermaker* or landscaper* or plumber* or waitress* or waiter* or fisherm*n or seam*n or sailor* or electrician* or (professional adj (sportsm*n or sportswom*n or athlete*))).ti,ab.
#5	#1 or #2 or #3 or #4
#6	exp meniscus/ or tibial meniscus injuries/ or menisc*.ti,ab.
#7	Knee/ and (Magnetic resonance imaging/ or arthroscopy/ or "International Classification of Diseases"/ or self report/ or (mri or magnetic resonance imag* or arthroscop* or (clinical adj (test* or examination*)) or self-report*).ti,ab.)
#8	Meniscectomy/ or knee injuries/ or (knee adj2 (pain or complaint* or injur* or disorder* or symptom*)).ti,ab.
#9	#6 or #7 or #8
#10	#5 and #9

2. Embase (via Elsevier): 28.02.2020

#1	Work/de or workplace/de or workload/de or employment/de or occupation/de or “occupational disease”/de or “occupational exposure”/de or “occupational hazard”/de or “occupational medicine”/de or “work environment”/de or “occupational health”/de or (occupation* or “work-related” or “working environment” or “work environment” or “work place*” or workplace* or “work site*” or worksite* or “work load” or workload or “at work” or worke* or job* or employment or industry):ti,ab
#2	“weight lifting”/de or “weight bearing”/de or “stair climbing”/de or “shear stress”/de or (lifting or carrying or kneeling or squatting or climbing or crawling or stooping or

	pivoting or twisting):ti,ab or (((running or moving or walking or jumping) near/2 (hard or uneven or rough or bumpy or slippery) near/2 (surface* or ground* or terrain*))):ti,ab or (knee near/1 (buckling or shifting or slipping)):ti,ab or ((shear* near/1 (stress* or force* or motion*))):ti,ab
#3	Mining/de or "coal mining"/de or gardening/de or agriculture/de or (mining or construction or tiling or shunting or paving or roofing or gardening or farming):ti,ab
#4	Miner/de or "agricultural worker"/de or "blue collar worker"/de or "construction worker"/de or "shipyard worker"/de or painter/de or carpenter/de or gardener/de or fisherman/de or sailor/de or electrician/de or "professional athlete"/de or (miner* or farmer* or tiler* or "tile setter*" or shunter* or "screed installer*" or "baggage handler*" or cleaner* or pipefitter* or woodworker* or labourer* or laborer* or bricklayer* or mason* or builder* or paver* or roofer* or slater* or painter* or decorator* or childcare or joiner* or carpenter* or "motor mechanic*" or "maintenance fitter*" or installer* or welder* or shipbuilder* or docker* or gardener* or millwright* or fitter* or boilermaker* or landscaper* or plumber* or waitress* or waiter* or fisherm*n or seam*n or sailor* or electrician*):ti,ab or (("blue-collar" or factory or "coal-face" or construction or road or concrete or shipyard or asphalt or metal) near/1 (worker* or labourer* or laborer*)):ti,ab or ((floor or carpet or parquet or pipe) near/1 (layer* or fitter*)):ti,ab or ((stove or oven) near/1 (maker* or builder* or fitter* or manufacturer*)):ti,ab or ((electrical or television or computer) near/1 engineer*):ti,ab or (professional near/1 (sportsm*n or sportswom*n or athlete*)):ti,ab
#5	#1 or #2 or #3 or #4
#6	„knee meniscus"/de or „knee meniscus rupture"/de or menisc*:ti,ab
#7	Knee/de and (("nuclear magnetic resonance imaging"/de or "international classification of diseases"/de or "self report"/de or "knee arthroscopy"/de or "clinical examination"/de) or (mri or "magnetic resonance imag*" or arthroscop* or "self-report*"):ti,ab or (clinical near/1 (test* or examination*)):ti,ab)
#8	Meniscectomy/de or "meniscal repair"/de or "meniscal transplantation"/de or "meniscal surgery"/de or "knee injury"/de or (knee near/2 (pain or complaint* or injur* or disorder* or symptom*)):ti,ab
#9	#6 or #7 or #8
#10	#5 and #9

3. Web of Science: 27.02.2020

#1	TS=(occupation* or "work-related" or "working environment" or "work environment" or workplace* or "work place*" or worksite* or "work site*" or workload or "work load" or "at work" or worke*or job* or employment or industry)<br>*Indexes=SCI-EXPANDED, SSCI, A&HCI Timespan=All years*
#2	TS=(((lifting or carrying) near/0 (load* or weight*)) or kneeling or climbing or squatting or crawling or stooping or ((running or moving or walking or jumping) near/2 ((hard or uneven or rough or bumpy or slippery) near/2 (surface* or ground* or terrain*))) or (knee near/0 (buckling or shifting or slipping)) or (shear* near/0 (stress* or force* or motion*) or pivoting or twisting))<br>*Indexes=SCI-EXPANDED, SSCI, A&HCI Timespan=All years*

#3	TS=(mining or gardening or construction or tiling or shunting or paving or roofing or farming)<br>*Indexes=SCI-EXPANDED, SSCI, A&HCI Timespan=All years*
#4	TS=(miner* or farmer* or (("blue-collar" or factory or "coal-face" or construction or road or concrete or shipyard or metal or asphalt) near/0 (worker* or laborer* or labourer*)) or ((floor or carpet or parquet or pipe) near/0 (layer* or fitter*)) or pipefitter* or woodworker* or tiler* or "tile setter*" or ((stove or oven) near/0 (maker* or builder* or fitter* or manufacturer*)) or shunter* or "screed installer*" or "baggage handler*" or bricklayer* or mason* or builder* or paver* or roofer* or slater* or painter* or decorator* or ((electrical or television or computer) near/0 engineer*) or cleaner* or laborer* or labourer* or childcare or joiner* or carpenter* or "motor mechanic*" or "maintenance fitter*" or installer* or welder* or shipbuilder* or docker* or gardener* or millwright* or fitter* or boilermaker* or landscaper* or plumber* or waitress* or waiter* or fisherm*n or seam*n or sailor* or electrician* or (professional near/0 (sportsm*n or sportswom*n or athlete*)))<br>*Indexes=SCI-EXPANDED, SSCI, A&HCI Timespan=All years*
#5	#4 OR #3 OR #2 OR #1<br>*Indexes=SCI-EXPANDED, SSCI, A&HCI Timespan=All years*
#6	TS=(menisc*)<br>*Indexes=SCI-EXPANDED, SSCI, A&HCI Timespan=All years*
#7	TS=(knee and ("magnetic resonance imag*" or mri or arthroscop* or "self report*" or (clinical near/0 (test* or examination*))))<br>*Indexes=SCI-EXPANDED, SSCI, A&HCI Timespan=All years*
#8	TS=(knee near/2 (pain or complaint* or injur* or disorder* or symptom*))<br>*Indexes=SCI-EXPANDED, SSCI, A&HCI Timespan=All years*
#9	#8 OR #7 OR #6<br>*Indexes=SCI-EXPANDED, SSCI, A&HCI Timespan=All years*
#10	#9 AND #5<br>*Indexes=SCI-EXPANDED, SSCI, A&HCI Timespan=All years*
