# Supplementary material for: Occupational risk factors for meniscal lesions: a systematic review and meta-analysis
Source: BMC Musculoskelet Disord. 2021 Dec 15;22:1042. doi: 10.1186/s12891-021-04900-7 (PMC8672613; doi:10.1186/s12891-021-04900-7)
Supplement: Supplementary file 2 — Additional file 2. Risk of bias schema. [file 12891_2021_4900_MOESM2_ESM.pdf]

Additional file 2: Risk of bias schema

Major risk of bias domains*	Risk	Criteria	Hints/ notes
**1. Recruitment procedure & follow-up (in cohort studies)**:<br><br>***For cohort studies***<br><br>*HINT: We are looking for selection bias:*<br>- *Was the cohort representative of a defined population? #*<br>- *Was everybody included who should have been included? #*<br>- *If response rate is slightly <50% but does not indicate selection bias, it will be listed as a demerit in extraction table.*<br><br>*PRELIMINARY RULING:*<br>- *If the cohort recruitment is based on a convenient/ self-reported sampling OR if response is <10% or not reported, the study will be excluded from analysis.*	low	☐ Cohort recruitment was acceptable.[#]<br>☐ Baseline response is acceptable (50% or more) OR is <50% and >30%, but substantial differential selection could be excluded.<br>☐ Loss to follow-up is below 20% in total and not different between the two groups (up to 10% difference).*	
	high	☐ Cohort recruitment was not acceptable.[#]<br>☐ Response not reported/ not calculable.<br>☐ Total loss to follow-up is larger than acceptable (20% or more)* OR drop out differs between the groups by more than 10%* OR the reasons for drop out considerably differ between exposed and non-exposed groups.*	
For case-control studies<br><br>*HINT: We are looking for selection bias:*<br>- *Were the cases and control subjects representative of the same defined population ("study base"; geographically and/or temporally)? #*<br>- *Was there an established reliable system for selecting all the cases? #*<br>- *The same exclusion criteria are used for both cases and controls. #*<br>- *Comparison is made between participants and non-participants to establish their similarities or differences. #*<br>- *If response rate is slightly <50% but does not indicate selection bias, it will be listed as a demerit in extraction table.*<br><br>*PRELIMINARY RULING:*<br>- *If the recruitment of the study population is based on a convenient/ self-reported sampling OR if response is <10% or not reported, the study will be excluded from analysis.*	low	☐ Case selection and recruitment was acceptable.[#]<br>☐ Control subjects' selection and recruitment was acceptable.[#]<br>☐ Non-response was less than 50% for cases and/or control subjects OR it was >50% and <70%, but substantial differential selection of cases and control subjects could be excluded*	
	high	☐ Case selection and recruitment was not acceptable.[#]<br>☐ Control subjects' selection and recruitment was not acceptable.[#]<br>☐ Non-response was >70% for cases or control subjects OR it was >50% and<70%, but substantial differential selection of cases and control subjects could not be excluded.*<br>☐ Response not reported/ not calculable	

*according to Ijaz et al. (2013), with modifications; # SIGN/CASP § Shamliyan

Major risk of bias domains*	Risk	Criteria	Hints/ notes
For cross-sectional studies<br>*HINT: We are looking for selection bias:*<br>- *Was the study population representative of a defined population? #*<br>- *Was everybody included who should have been included? #*<br>- *If response rate is slightly <50% but does not indicate selection bias, it will be listed as a demerit in extraction table.*<br>*PRELIMINARY RULING:*<br>- *If the recruitment of the study population is based on a convenient/ self-reported sampling OR if response is <10% or not reported, the study will be excluded from analysis.*	low	☐ Recruitment of the study population was acceptable.[#]<br>☐ Non-response was less than 50% OR it was >50% and <70%, but substantial differential selection of the study population could be excluded.*	
	high	☐ Recruitment of the study population was not acceptable.[#]<br>☐ Non-response was >70% OR it was >50% and <70%, but substantial differential selection of the study population could not be excluded.*<br>☐ Response not reported/ not calculable.	
**2. Exposure definition and measurement**	low	☐ Exposure was defined adequately covering more than one aspect of exposure (duration, frequency, intensity) and was assessed objectively: direct measurement or systematic observations or using a questionnaire that is validated.*	
	high	☐ Exposure was not defined adequately covering only one aspect of exposure (duration, frequency, intensity) and/or was assessed subjectively (self-report, questionnaire, interview) or using a proxy used to allocate exposure status (job matrix, job title).*<br>☐ Different methods were used to measure exposure in different groups/ cases and control subjects (***in case-control studies***).[§]	
	unclear	☐ Not reported.	
**3. Outcome "rate of/ risk to develop meniscal lesions". Source and validation**	low	☐ Outcome was accurately/ objectively measured to minimize bias (e.g. arthroscopically, MRI, open surgery)[#]<br>☐ Measurement methods were similar in the different groups.[#]	
	high	☐ Outcome was not accurately or subjectively measured (self-reported, clinical examination).[#]<br>☐ Measurement methods were different in the groups.[#]	
	unclear	☐ Not reported.	
**4. Confounding and effect modification**	low	☐ If risk estimators were calculated, major confounding factors (age, sex) were considered.<br>☐ If only prevalence or incidence was assessed, at least sex and age are described.	
	high	☐ Major confounding factors (age, sex) were not considered.	
	unclear	☐ Not reported.	

*according to Ijaz et al. (2013), with modifications; # SIGN/CASP § Shamliyan

Major risk of bias domains*	Risk	Criteria	Hints/ notes
**5. Analysis method: methods to reduce research specific bias**	low	☐ Authors used adequate statistical models to reduce bias (e.g. standardization, matching, adjustment in multivariate model, stratification, propensity scoring).§	
	high	☐ Authors did not use adequate statistical models to reduce bias.	
	unclear	☐ Not reported.	
**6. Chronology**	low	☐ Incident diseases were included.#<br>☐ Temporal relation may be established (exposure precedes the outcome).#<br>☐ No meniscal damage known at baseline ***(in cohort and case-control-studies).***	
	high	☐ People with prevalent meniscal damage were included OR people with prevalent meniscal damage of baseline were not excluded ***(in cohort studies)***.#<br>☐ Temporal relation cannot be established.<br>☐ Meniscal status is unknown at baseline.	
	unclear	☐ Not reported.	

Minor risk of bias domains*	Risk	Criteria	Hints/ notes
**7. Blinding of assessors**	low	☐ Assessors were reported or indicated to be blind for individual exposure-status in cohort and cross-sectional studies and to case status in case-control and cross-sectional studies	
	high	☐ Assessors were reported or indicated <u>not</u> to be blind for individual exposure-status in cohort and cross-sectional studies and to case status in case-control and cross-sectional studies	
	unclear	☐ Not reported.	
**8. Funding**	low	☐ Grant/ non-profit-organizations*<br>☐ Study was clearly not affected by sponsors.*	
	high	☐ Sponsoring organization participated in data analysis.<br>☐ Study was probably affected by sponsors.	
	unclear	☐ Industry, combined industry+grant*, unclear if study was affected by sponsors.<br>☐ Not reported.	
**9. Conflict of interest**	low	☐ Reported not having conflict of interest or clear from report/ communication that study was not affected by author(s) affiliation.*	
	high	☐ Conflict of interest exists (at least one author).*	
	unclear	☐ Not reported.	

*according to Ijaz et al. (2013), with modifications; # SIGN/CASP § Shamliyan

Overall risk of bias assessment		Low Risk	High Risk	Unclear Risk
Major domains	1. Recruitment procedure & follow-up (in cohort studies)			
	2. Exposure definition and measurement			
	3. Outcome “rate of/ risk to develop meniscal lesions”. Source and validation			
	4. Confounding and effect modification			
	5. Analysis method: methods to reduce research specific bias			
	6. Chronology			
Minor domains	7. Blinding of assessors			
	8. Funding			
	9. Conflict of interest			
**General rule for rating:**	**Low risk of bias:** low risk in all major domains<br>**High risk of bias:** if not low risk<br>**Overall assessment:**			

*according to Ijaz et al. (2013), with modifications; # SIGN/CASP § Shamliyan
