# Supplementary material for: Occupational risk factors for meniscal lesions: a systematic review and meta-analysis
Source: BMC Musculoskelet Disord. 2021 Dec 15;22:1042. doi: 10.1186/s12891-021-04900-7 (PMC8672613; doi:10.1186/s12891-021-04900-7)
Supplement: Supplementary file 3 — Additional file 3. List of excluded studies. [file 12891_2021_4900_MOESM3_ESM.pdf]

Additional file 3: List of excluded studies (listed alphabetically)

Study	Reason for exclusion
Alizai et al., 2012 [1]	Conference paper to publication from Virayavanich et al. (2013).
Atkins, 1957 [2]	No epidemiological observational study.<br>Not related to a population.
Beaulieu-Jones et al., 2017 [3]	No working population – collegiate football players are amateurs.
Boeth et al., 2017 [4]	No response-rate reported.
Bonar, 1950 [5]	No response-rate reported.
Brunner, 1989 [6]	No response-rate reported.
Cha et al., 2015 [7]	No response-rate reported.<br>No prevalence data on meniscal lesions – outcome cartilage of the ankle.
Chahla et al., 2018 [8]	No prevalence data on meniscal lesions reported as outcome.
Dale et al., 2015 [9]	No prevalence data on meniscal lesions reported as outcome.
De Brouwer & Lecomte, 1996 [10]	No response-rate reported.<br>Prevalence data of meniscal lesions were not assigned to individual occupational groups.
Deitch et al., 2006 [11]	Injury-related meniscal lesions reported as outcome.
Dorè et al., 2012 [12]	No work-related exposure.
Duman et al., 2014 [13]	Conference abstract, no full text available.
Etz, 1999 [14]	Prevalence data of meniscal lesions were not assigned to individual occupational groups.
Fencolvá et al., 2005 [15]	No primary data. Secondary analysis of data from occupational diseases statistics.
Greinemann, 1983 [16]	No response-rate reported in controls.
Greinemann, 1988 [17]	No response-rate reported.
Harkensee & Hillebrandt, 2019 [18]	No prevalence data on meniscal lesions reported as outcome.
Hartman & Seidel, 2003 [19]	No prevalence data on meniscal lesions reported as outcome.
Heimel & Hatting, 1982 [20]	No epidemiological observational study design.
Hess, 1975 [21]	Not related to a population.<br>No response-rate reported.
Hill et al., 2013 [22]	Injury-related meniscal lesions reported as outcome.
Holibkova et al., 1989 [23]	Not related to a population.
Hwang et al., 2012 [24]	No work-related exposure.<br>Not related to a population.
Jensen et al., 2000 [25]	No prevalence data on meniscal lesions reported as outcome.
Jones et al., 2012 [26]	Injury-related meniscal lesions reported as outcome.
Kettunen et al., 2001 [27]	Injury-related meniscal lesions reported as outcome.
Kolms, 1980 [28]	Meniscal lesions after occupational accidents.
Kompel et al., 2018 [29]	Injury-related meniscal lesions reported as outcome.
Krinsky et al., 1992 [30]	Injury-related meniscal lesions reported as outcome.
Kuikka et al., 2013 [31]	No working population – no professional soldiers but recruits with average exposure duration of 9 months.

Larsen et al., 1999 [32]	Injury-related meniscal lesions reported as outcome.
Major & Helms, 2002 [33]	No working population – collegiate basketball players are amateurs.
Matiotti et al., 2017 [34]	No response-rate reported.<br>No working population – adolescent soccer players aged 14-17 years.
McCarthy et al., 2013 [35]	Injury-related meniscal lesions reported as outcome.
Mikes, 1985 [36]	Not related to a population.
Nauwald, 1975 [37]	No prevalence data on meniscal lesions reported as outcome.
Nepple et al., 2012 [38]	No working population – collegiate football players are amateurs.
Newman & Newberg, 2010 [39]	Systematic review.
Pearce et al., 1996 [40]	Prevalence data of meniscal lesions were not assigned to individual occupational groups.
Prien et al., 2017 [41]	No prevalence data on meniscal lesions reported as outcome.
Puntumetakul et al., 2018 [42]	No response-rate reported.
Schneider, 1975 [43]	No clinical study.
Schram et al., 2018 [44]	No prevalence data on meniscal lesions reported as outcome.
Sharifi et al., 2017 [45]	Not related to a population.
Sharrard, 1965 [46]	Reports the same data as Sharrard & Liddell (1962).
Shellock et al., 1991	No work-related exposure (amateur athletes).
Shin et al., 2016 [47]	No response-rate reported.
Soder et al., 2011 [48]	No response-rate reported.<br>No working population – adolescent soccer players aged 14 or 15 years.
Spector et al., 2011 [49]	No prevalence data on meniscal lesions reported as outcome.
Springorum, 1968 [50]	Not related to a population.
Springorum, 1969 [51]	Not related to a population.<br>No response-rate reported.
Thelin et al., 2006 [52]	No prevalence data on meniscal lesions – main outcome osteoarthritis.<br>No separate analysis for sports at competition level.
Uher, 1968 [53]	Not related to a population.
Virayavanich et al., 2013 [54]	No response-rate reported.
Wacker, 1995 [55]	No response rate reported.<br>No working population – adolescent athletes aged 12-13 years.
Yeh et al., 2012 [56]	No information on how meniscal lesions were assessed.
Yuan et al., 2011 [57]	No information on how meniscal lesions were assessed.
Ziolkiewicz & Gazdzik, 2000 [58]	No response-rate reported.<br>Not related to a population.

1. Alizai H, Virayavanich W, Lin W, Nardo L, Nevitt MC, Lynch JA, et al. Association of frequent knee bending activity with focal knee lesions detected with 3T MRI over a period of 3 yearsdata from the osteoarthritis initiative. Osteoarthritis and Cartilage. 2012;20:S213.
2. Atkins JB. Internal derangement of the knee joint in miners. British journal of industrial medicine. 1957;14(2):121-6.
3. Beaulieu-Jones BR, Rossy WH, Sanchez G, Whalen JM, Lavery KP, McHale KJ, et al. Epidemiology of Injuries Identified at the NFL Scouting Combine and Their Impact on Performance in the National Football League: Evaluation of 2203 Athletes From 2009 to 2015. Orthopaedic journal of sports medicine. 2017;5(7):2325967117708744-.
4. Boeth H, MacMahon A, Eckstein F, Diederichs G, Schlausch A, Wirth W, et al. MRI findings of knee abnormalities in adolescent and adult volleyball players. J Exp Orthop. 2017;4(1):6.
5. Bonar AA. Injuries of the semilunar cartilages in miners: a review of 200 cases, with special reference to the post-operative disability time. Glasgow medical journal. 1950;31(6):197-205.
6. Brunner MC, Flower SP, Evancho AM, Allman FL, Apple DF, Fajman WA. MRI of the athletic knee. Findings in asymptomatic professional basketball and collegiate football players. Investigative radiology. 1989;24(1):72-5.
7. Cha JG, Yi JS, Han JK, Lee YK. Comparison of Quantitative Cartilage T2 Measurements and Qualitative MR Imaging between Professional Ballet Dancers and Healthy Volunteers. Radiology. 2015;276(1):199-206.
8. Chahla J, Cinque ME, Godin JA, Sanchez G, Lebus GF, Whalen JM, et al. Meniscectomy and Resultant Articular Cartilage Lesions of the Knee Among Prospective National Football League Players: An Imaging and Performance Analysis. The American journal of sports medicine. 2018;46(1):200-7.
9. Dale AM, Ryan D, Welch L, Olsen MA, Buchholz B, Evanoff B. Comparison of musculoskeletal disorder health claims between construction floor layers and a general working population. Occupational and Environmental Medicine. 2015;72(1):15-20.
10. De Brouwer C, Lecomte A. Epidemiologic study of knee pain in a hospital. Archives des Maladies Professionnelles et de Medecine du Travail. 1996;57(3):168-75.
11. Deitch JR, Starkey C, Walters SL, Moseley JB. Injury risk in professional basketball players: A comparison of Women's National Basketball Association and National Basketball Association athletes. American Journal of Sports Medicine. 2006;34(7):1077-83.
12. Dorè D, Winzenberg T, Ding C, Otahal P, Pelletier J, Martel-Pelletier J, et al. The association between objectively measured physical activity and knee structural change using magnetic resonance imaging (MRI). Journal of Science and Medicine in Sport. 2012;15:S243-S4.
13. Duman F, Doǃru E, Canbay Ö, Yücekaya B, Duman T, Korkmaz NÇ, et al. Investigation ot knee problems incidence in security guards. Fizyoterapi Rehabilitasyon. 2014;25(1):S88-S9.
14. Etz P. Unfitness for work as a result of disease and injury of the knee joint. Arbeitsmedizin Sozialmedizin Umweltmedizin. 1999;34(9):380-6.
15. Fenclová Z, Urban P, Pelclová D, Lebedová J, Lukáš E. Analysis of incidence of diseases from excessive load upon extremities as notified in the Czech Republic during 1996-2003. Ceske Pracovni Lekarstvi. 2005;6(1):15-21.
16. Greinemann H. Prädestinieren Kniescheibenhochstand, Knie- und Kniescheibenfehlformen sowie Beinachsenfehlstellungen bei kniebelastenden Berufen zu vorzeitigen Verschleißschäden? Forschungsbericht Nr. 362 der Bundesanstalt für Arbeitsschutz. Bremerhaven: Wirtschaftsverlag NW; 1983.
17. Greinemann H. [Arguments against the recognition of arthroses of the knee joint following occupational stress as an occupational disease]. Unfallchirurg. 1988;91(8):374-80.
18. Harkensee C, Hillebrandt D. An Occupational Health Survey of British Mountain Guides Operating Internationally. Wilderness & environmental medicine. 2019;30(3):236-43.
19. Hartmann B, Seidel D. The knee joint under the physical stresses of the construction industry. Zentralblatt fur Arbeitsmedizin, Arbeitsschutz und Ergonomie. 2003;53(8):428-41.
20. Heimel R, Hatting W. [Results of a long-term study of total meniscectomy in coal-miners with degenerative meniscus damage (author's transl)]. Die Ergebnisse der totalen Menisektomie bei

Bergleuten mit anerkannter Berufserkrankung Eine vergleichende Langzeitstudie an 3021 Fallen. 1982;85(1):25-9.
21. Hess H. [Degenerative meniscus lesions in occupational soccer players--an occupational disease?]. Der degenerative Meniskusschaden der Berufsfussballspieler-eine Berufskrankheit. 1975;113(4):669-72.
22. Hill OT, Bulathsinhala L, Scofield DE, Haley TF, Bernasek TL. Risk factors for soft tissue knee injuries in active duty U.S. Army soldiers, 2000-2005. Military medicine. 2013;178(6):676-82.
23. Holibkova A, Machalek L, Holibka R, Chromek Z. Lesions of the knee joint menisci in miners. Acta Universitatis Palackianae Olomucensis Facultatis Medicae. 1989;123:147-68.
24. Hwang B-Y, Kim S-J, Lee S-W, Lee H-E, Lee C-K, Hunter DJ, et al. Risk factors for medial meniscus posterior root tear. The American journal of sports medicine. 2012;40(7):1606-10.
25. Jensen LK, Mikkelsen S, Loft IP, Eenberg W, Bergmann I, Logager V. Radiographic knee osteoarthritis in floorlayers and carpenters. Scandinavian journal of work, environment & health. 2000;26(3):257-62.
26. Jones JC, Burks R, Owens BD, Sturdivant RX, Svoboda SJ, Cameron KL. Incidence and risk factors associated with meniscal injuries among active-duty US military service members. Journal of athletic training. 2012;47(1):67-73.
27. Kettunen JA, Kujala UM, Kaprio J, Koskenvuo M, Sarna S. Lower-limb function among former elite male athletes. The American journal of sports medicine. 2001;29(1):2-8.
28. Kolms D. Erkrankungen und Verletzungen der Kniegelenke verschiedener Tätigkeitsgruppen mit erhöhter Kniegelenksbelastung ubB Meniskusläsion: eine arbeitsmedizinische Untersuchung [Doctoral dissertation]. Frankfurt am Main,1980.
29. Kompel AJ, Murakami AM, Engebretsen L, Forster BB, Lotfi M, Jarraya M, et al. MRI-Detected Sports-Related Knee Injuries and Abnormalities at the Rio de Janeiro 2016 Summer Olympic Games. American Journal of Roentgenology. 2018;211(4):880-6.
30. Krinsky MB, Abdenour TE, Starkey C, Albo RA, Chu DA. Incidence of lateral meniscus injury in professional basketball players. The American journal of sports medicine. 1992;20(1):17-9.
31. Kuikka PI, Pihlajamäki HK, Mattila VM. Knee injuries related to sports in young adult males during military service - incidence and risk factors. Scand J Med Sci Sports. 2013;23(3):281-7.
32. Larsen E, Jensen PK, Jensen PR. Long-term outcome of knee and ankle injuries in elite football. Scandinavian journal of medicine & science in sports. 1999;9(5):285-9.
33. Major NM, Helms CA. MR imaging of the knee: findings in asymptomatic collegiate basketball players. AJR Am J Roentgenol. 2002;179(3):641-4.
34. Matiotti SB, Soder RB, Becker RG, Santos FS, Baldisserotto M. MRI of the knees in asymptomatic adolescent soccer players: A case-control study. J Magn Reson Imaging. 2017;45(1):59-65.
35. McCarthy MM, Voos JE, Nguyen JT, Callahan L, Hannafin JA. Injury profile in elite female basketball athletes at the Women's National Basketball Association combine. The American journal of sports medicine. 2013;41(3):645-51.
36. Mikes K. [Incidence of meniscal lesions in persons working underground in comparison with the rest of the population]. Vyskyt lezi menisku u pracujicich v podzemi ve srovnani s ostatni populaci. 1985;52(2):138-41.
37. Nauwald G. [Incidence of diseases of the knee joint in the orthopedic-industrial medicine care of workers in the ship-building industry with regard to some industrial medicine aspects]. Uber die Haufigkeit von Kniegelenkserkrankungen in der orthopadisch-arbeits-medizinischen Betreuung von Werkern der Schiffbauindustrie unter Berucksichtigung einiger arbeitsmedizinischer Aspekte. 1975;69(23):1235-41.
38. Nepple JJ, Dunn WR, Wright RW. Meniscal repair outcomes at greater than five years: A systematic literature review and meta-analysis. Journal of Bone and Joint Surgery - Series A. 2012;94(24):2222-7.
39. Newman JS, Newberg AH. Basketball Injuries. Radiologic Clinics. 2010;48(6):1095-111.
40. Pearce MS, Buttery YE, Brueton RN. Knee Pathology among Seafarers: A Review of 299 Patients. Occupational Medicine. 1996;46(2):137-40.

41. Prien A, Prinz B, Dvořák J, Junge A. Health problems in former elite female football players: Prevalence and risk factors. Scandinavian Journal of Medicine & Science in Sports. 2017;27(11):1404-10.
42. Puntumetakul R, Neubert MS, Karukunchit U, Buranruk O, Boucaut R. Knee musculoskeletal impairments and associated pain factors among rice farmers. Journal of back and musculoskeletal rehabilitation. 2018;31(6):1111-7.
43. Schneider PG. [Meniscus lesions in occupational soccer players]. Meniskusschaden bei Berufsfussballern. 1975;113(4):666-8.
44. Schram B, Orr R, Pope R. A Profile of Knee Injuries Suffered by Australian Army Reserve Soldiers. International journal of environmental research and public health. 2018;16(1).
45. Sharifi K, Mahmoodi M, Abdolmohammadi J, Moradi B, Karami S, Bromandi K, et al. Investigation of the frequency of meniscal ligament injuries and other abnormal knee injuries in the patients referring to MRI center of Shahid Ghazi Clinic in Sanandaj. Scientific Journal of Kurdistan University of Medical Sciences. 2017;21(6):55-63.
46. Sharrard WJ. PRESSURE EFFECTS ON THE KNEE IN KNEELING MINERS. Annals of the Royal College of Surgeons of England. 1965;36:309-24.
47. Shin D, Youn K, Lee E, Lee M, Chung H, Kim D. Risk factors for lesions of the knee menisci among workers in South Korea's national parks. Annals of Occupational and Environmental Medicine. 2016;28(1).
48. Soder RB, Simões JD, Soder JB, Baldisserotto M. MRI of the knee joint in asymptomatic adolescent soccer players: a controlled study. AJR Am J Roentgenol. 2011;196(1):W61-5.
49. Spector JT, Adams D, Silverstein B. Burden of work-related knee disorders in Washington State, 1999 to 2007. Journal of occupational and environmental medicine. 2011;53(5):537-47.
50. Springorum PW. [Occupational meniscus lesions in other persons than miners]. Berufliche Meniscusschaden ausserhalb des Bergbaus. 1968;71(7):288-94.
51. Springorum PW. [Influence of working conditions on meniscus damage in miners]. Der Einfluss der Arbeitsweise auf Meniscusschaden bei Bergleuten. 1969;72(11):477-81.
52. Thelin N, Holmberg S, Thelin A. Knee injuries account for the sports-related increased risk of knee osteoarthritis. Scandinavian journal of medicine & science in sports. 2006;16(5):329-33.
53. Uher M. [Non-traumatic ruptures of the knee joint menisci in miners]. Neurazove ruptury menisku kolennich kloubu u horniku. 1968;35(3):260-2.
54. Virayavanich W, Alizai H, Baum T, Nardo L, Nevitt MC, Lynch JA, et al. Association of frequent knee bending activity with focal knee lesions detected with 3T magnetic resonance imaging: data from the osteoarthritis initiative. Arthritis care & research. 2013;65(9):1441-8.
55. Wacker F, Bolze X, Mellerowicz H, Wolf KJ. [Diagnosis of changes in the knee joint of high performance athletes]. Diagnostik von Veranderungen des Kniegelenkes bei Leistungssportlern. 1995;35(2):94-100.
56. Yeh PC, Starkey C, Lombardo S, Vitti G, Kharrazi FD. Epidemiology of isolated meniscal injury and its effect on performance in athletes from the National Basketball Association. The American journal of sports medicine. 2012;40(3):589-94.
57. Yuan L. Knee Disorders Among Carpenters in the St. Louis Area. The Open Occupational Health & Safety Journal. 2011;3:31-8.
58. Ziolkiewicz M, Gazdzik TS. [Characteristics of intra-articular injuries of the knee joint in coal miners]. Charakterystyka uszkodzen wewnatrzstawowych powstalych w nastepstwie przewleklych urazow stawu kolanowego u gornikow. 2000;65(6):651-6.
