# Supplementary material for: Occupational risk factors for meniscal lesions: a systematic review and meta-analysis
Source: BMC Musculoskelet Disord. 2021 Dec 15;22:1042. doi: 10.1186/s12891-021-04900-7 (PMC8672613; doi:10.1186/s12891-021-04900-7)
Supplement: Supplementary file 4 — Additional file 4. Study characteristics of included studies. [file 12891_2021_4900_MOESM4_ESM.docx]

**Additional file 4:** Study characteristics of included studies

Study characteristics of included cohort and cross-sectional studies

| Study | Study region | Study design | Time of recruitment/ follow-up | Population  *Source/setting, number of participants, age, gender*  *occupational exposure (definition, job title, duration, method to identify exposure)* | | Outcome  *definition, measurement* |
| --- | --- | --- | --- | --- | --- | --- |
|  |  |  |  | Exposure group | Comparison group |  |
| Behzadi et al. (2017) | Federal Republic of Germany | Cross-sectional study | NR | Elite professional football players from a team of the 1. German Bundesliga were examined during a routine medical check-up prior to contract enrollment or pre-season training.  n=22  **Age, years (mean, SD (range))**  23.47, 2.68 (18-26)  **Gender**  All male  **Response**  No information, presumably 100% because of the nature of the study prior to contract or pre-season training  **Exposure intensity**  Playing in the 1. German football league  **Exposure duration**  NR | Healthy amateur athletes recruited from local medical students who routinely perform 120 to 180 min of sports per week at an amateur level  n=22  **Age, years (mean, SD (range))**  24.11, 1.96 (18-26)  **Gender**  All male  **Response**  NR | Meniscal lesion, meniscal degeneration diagnosed in magnetic resonance image at 3 Tesla.  Meniscal lesions were classified according to Stoller et al. (1987). Meniscal degeneration was categorized into three grades according to Cheung et al. (1997). |
| Bezuglov et al. (2019) | Russia | Cross-sectional study | December 2014 – January 2019 | Professional male elite soccer players who underwent medical examination before signing a contract with the leading Russian Premier League soccer clubs.  n=47 (94 knee joints)  **Age, years (mean, SD)**  25.7, 4.6  **Gender**  All male  **Response**  100%  **Exposure intensity**  Playing football on professional level. All players had been or were members of junior or adult national teams of their countries.  **Exposure duration**  All the participants had played soccer for 6 to 7 years and had performed in 80 or more matches as members of professional leagues of their countries. No information according the duration of the professional career. | No controls were screened. | Presence of meniscal lesions assessed through 1.5-T MRI scanners.  Meniscal lesions were graded separately with the system described by Stoller et al. (1987). |
| Brouwer et al. (1981) | Netherlands | Cross-sectional study | 1978 | Former professional soccer players in the highest leagues in the Netherland in the year 1956 in the province of Limburg.  n=43  **Age, years (mean (range))**  45.9 (37-55)  **Gender**  NR, presumably all male because the first professional women’s league (Eredivisie) was established in 2007.  **Response**  60%  **Exposure intensity**  Participants played football on professional level in the year 1956.  **Exposure duration**  The participants had started to play competitive football at the age of about 16 years. Their careers as juniors, amateurs and professionals lasted on average 20 years. Participants were playing football for 4-10 hours per week. | Healthy persons with comparable age who had never played competitive football were selected from a random sample of officials of the municipality of Maastricht, Limburg.  n=43  **Age, years (mean (range))**  46.8 (38-55)  **Gender**  NR, presumably all male  **Response**  41% | Self-reported meniscectomy assessed through questionnaire. |
| Hong et al. (2020) | South Korea | Cross-sectional study | 2013 – 2015 | Farmers registered in the Korea Farmer’s Knee Cohort in 2013-2015.  n=486 (total)  n=330 workers with moderate or high exposure to heavy-lifting (≥2,000 hours)  n=248 workers with moderate or high exposure to squatting (≥10,000 hours)  **Age, years (mean, SD (range))**  56.1, 7.2 (40-69)  without differentiation according working exposure  **Gender (n (%))**  Female: 248 (51.0)  Male: 238 (49.0)  without differentiation according working exposure  **Response**  88%  **Exposure intensity**  Heavy-lifting and squatting  **Exposure duration**  Cumulative heavy-lifting working time (CLWT), hours (mean, SD): 16574.5, 20669.2  <2,000 hours (n (%)): 156 (32.1)  2,000-4,999 hours (n (%)): 59 (12.1)  ≥5,000 hours (n (%)): 271 (55.8)  Cumulative squatting working time (CSWT), hours (mean, SD): 20473.2, 23084.9  <10,000 hours (n (%)): 238 (49.0)  10,000-19,999 hours (n (%)): 64 (13.1)  ≥20,000 hours (n (%)): 184 (37.9) | Farmers with low exposure to heavy-lifting or squatting.  n=156 workers with low exposure to heavy-lifting (<2,000 hours)  n=238 workers with low exposure to squatting (<10,000 hours)  **Age, gender, response: see left** | Presence of meniscal lesions in both knees was assessed through 1.5 Tesla MRI scanner.  Meniscal lesions were classified as per the MRI Osteoarthritis Knee Score classification system for meniscal tears (Hunter et al. (2011)). |
| Kaplan et al. (2005) | USA | Cross-sectional study | July 1996 – August 1999 | Male professional basketball players who had MRI examination of both knees as part of their preseason evaluation. Players needed full painless range of motion, no joint-line tenderness, negative patella femoral compression testing, and no neurovascular compromise.  n=20  **Age, years (mean (range))**  26.2 (21-36)  **Gender**  All male  **Response**  NR, presumably 100 % because of the nature of the study as preseason evaluation of professional players.  **Exposure intensity**  Playing basketball on professional level.  **Exposure duration**  NR | No controls were screened. | Grade 1-3 meniscus lesions according of Crues et al. (1987) (identical as in Stoller et al. (1987)) in a 1.5-Tesla MRI of both knees. |
| Kivimäki et al. (1992) | Finland | Cross-sectional study | NR | Actively working male floor and carpet layers who ranged in age from 25 to 49 years, lived in southern Finland, had at least five years of experience in their current occupation, and were registered members of the trade union were enlisted for the study.  n=168  **Age, years (mean, SD)**  38.5, 6.8  **Gender**  All male  **Response**  78%  **Exposure intensity**  Kneeling with one or both knees on the floor (42% of total observation time); Squatting with knees flexed more than 90 degrees (3% of the total observation time)  **Exposure duration**  Average working time in the present occupation, years (mean, SD): 14.7, 7.7  Average worktime: 40h/week | House painters were selected with the same criteria (see left) with the use of frequency matching according to five-year age strata.  n=146  **Age, years (mean, SD)**  39.1, 6.7  **Gender**  All male  **Response**  66%  **Exposure intensity**  Kneeling with one or both knees on the floor (<3% of total observation time); Squatting with knees flexed more than 90 degrees (3% of the total observation time)  **Exposure duration (mean, SD)**  18.1, 7.5 | Self-reported meniscal lesions (verified by a physician) assessed in a standardized questionnaire. No information whether the medial or lateral meniscus was involved. |
| Kontio et al. (2017) | Finland | Cohort study | 1 January 1978 – 31 December 2010^1^ | A population sample of 8000 subjects (3637 men and 4363 women) was drawn from the population register to represent Finnish adults aged 30 years or over. A systematic sample was drawn from 40 representative geographical areas (Mini-Finland health survey).  n=4713 (total)  n=1043 workers with heavy physical strenuousness of work  n=1954 workers with moderate physical strenuousness of work  **Age, years**  30-39 year: n=1,815  40-49 year: n=1,541  50-59 year: n=1,357  without differentiation according physical strenuousness of work  **Gender (n (%))**  Female: 2393 (50.8)  Male: 2320 (49.2)  without differentiation according physical strenuousness of work  **Response**  93% at the initial screening phase in 1978-1980 without differentiation according physical strenuousness of work. 100 % according the follow-up registration of meniscus lesions by record-linkage.  **Exposure intensity**  Heavy strenuousness of work:  Not defined in this publication - According to Heliövaara et al. (1993) strenuousness of work was assessed in this study by a questionnaire according lifting and carrying heavy objects, stooped, twisted or otherwise awkward work postures, in intensity vibration of the whole body or use of vibrating equipment, a continuously repeated series of movements, and working speed determined by a machine.  Moderately heavy work: including standing and some walking  **Exposure duration**  NR | Mini-Finland health survey with workers with light physical strenuousness of work or mostly sedentary work.  n=1264  **Age, gender, response: see left** | Registration of incident hospitalizations due to acute and chronic meniscal lesions with the national hospital discharge register.  The following ICD codes (ICD-8, ICD-9, ICD-10) were used to identify and classify the first meniscal lesion: 724,10; 7170A – 7179X; 8360A; 8361A; 8362A; M23.0 –  M23.9; S83.2. |
| Krajnc et al. (2010) | Slovenia | Cross-sectional study | NR | Former football players from the football club NK Maribor who had played at a professional level in the first Slovenian league.  n=40  **Age, years (mean, SD)**  49.2, 9.7  **Gender**  NR, presumably all male, since there is no professional soccer league for women in Slovenia  **Response**  87%  **Exposure intensity**  Playing football on professional level.  Playing position (n (%)):  Goalkeeper: 7 (17.5)  Defender: 12 (30.0)  Midfielder: 10 (25.0)  Forward: 11 (27.5)  **Exposure duration**  Total duration of football career, years (mean, SD): 18.9, 3.8  Duration of playing in the first Slovenian football league (≥ 3 years), years (mean, SD): 11.3, 4.2 | No controls were screened. | Self-reported meniscus surgeries assessed through standardized questionnaire. |
| Mikkelsen et al. (2016) | Denmark | Cohort study | January 1990 – 31 December 2012^1^ | Baggage handlers from electronic employee files of two baggage handling companies at Copenhagen Airport and from electronic member files of the local union of unskilled workers that organized baggage handlers in the airport.  n=3.307  **Age, years (n (%))**  <30: 2.044 (62)  30-44: 1.151 (35)  45-59: 110 (3)  60+: 2 (0)  **Gender**  All male  **Response**  100% in the record-linkage and 68.4% in the questionnaire study 2012  **Exposure intensity**  Baggage sorting area: Loading or unloading baggage pieces (average weight 15 kg) from baggage carts and baggage containers. Pneumatic lifting hooks were introduced in 1998 and were used most of the time in 34%, sometimes in 37%, and seldom in 24% of workers according a questionnaire study in 3,092 baggage handlers in 2012.  Working on the apron: Loading or unloading baggage pieces from baggage carts to or from a belt conveyer to the baggage compartment opening of the aircraft that is height adjustable or work in the aircraft baggage compartment of the aircraft with handling of baggage pieces in a standing, stooped, sitting, squatting or kneeling work position. During 2002-2004 flexible belt loaders at the aircrafts were introduced. The baggage handlers lifted approximately 5 tons of baggage pieces during a 9-h workday.  Two measures of exposure were included: work as a baggage handler (ever: yes/no) and cumulative years of employment as a baggage handler.  **Exposure duration**  0.1-2.9 years: 86/277 (31.0), 3.0-9-9 years: 107/277 (38.6), 10.0-19.9 years: 66/277 (23.8), ≥20 years: 18/277 (6.5) | Former and present unskilled  workers with a variety of different tasks within the airport (e.g. guards and security personnel, area maintenance, cleaning, firefighting) and outside the airport but within the greater Copenhagen area (e.g. municipal workers, drivers, postal workers, garbage collectors, factory workers).  n=63.934  **Age, years (n (%))**  <30: 29.605 (46)  30-44: 20.597 (32)  45-59: 9.888 (15)  60+: 3.844 (6)  **Gender**  All male  **Response**  Response rate: 100% in the record-linkage and 68.5% in the questionnaire study 2012 | First hospitalization with a meniscal disease as the primary discharge diagnosis or surgery for a meniscal lesion.  Diagnoses and surgical procedures as meniscal lesions: ICD-8:  Old meniscal disease (724.19), knee distorsion with meniscal lesions (844.02, 844.03, 844.04),  traumatic lesion of menisci (849.45, 849.46, 849.48, 849.49); ICD-10: old traumatic meniscal  lesion (M23.2), other meniscal disorders (M23.3), traumatic meniscal rupture (S83.2) and traumatic  lesion of menisci and ligaments (S83.7C); Danish surgical procedure classification codes  before 1996: resection of meniscus (72540, 72541, 72549, 74560, 74570), meniscectomy (72640,  72641, 72649, 74580, 74590,74600, 74610), reinsertion of meniscus (72740, 72741, 72749,  74620, 74630); Nomesco-codes from 1996: operations on menisci of the knee (NGD-group).  From the basic cohort persons with an outcome before first date of employment were excluded. |
| Musialek & Kostal (1995) | Czech Republic | Cross-sectional study | 1988-1989^1^ | Miners from the region of Ostrava (total 19,170 miners), Czech Republic, who were admitted to hospital due to meniscal lesions and treated with open surgery. Only miners working underground with surgically verified diagnosis of meniscal lesion and complete medical documentation were included.  There is no information according the kind of mining. Certainly, the study was made in hard coal miners, because Ostrava is famous for this industry.  n=98  **Age, years (mean)**  34.1  **Gender**  All male  **Response**  NR, but presumably 100% because of the nature of the study as retrospective analysis of hospital records.  **Exposure intensity**  Working as an underground coal miner. No further information, e.g. face work or exposure to kneeling or squatting.  **Exposure duration**  NR | Persons not working as miners from the region of Ostrava (total 310,000 inhabitants), Czech Republic, who were admitted to hospital due to meniscal lesions and treated with open surgery.  n=192  **Age, years (mean)**  35.2  **Gender**  All male  **Response**  See left | Incidence of meniscal lesions treated with open surgery. |
| Nauwald (1980) | German Democratic Republic | Cross-sectional study | NR | Manual welders in shipbuilding.  n=100  **Age, years (n (%))**  ≤ 25 years: 9 (9.0)  26-35 years: 28 (28.0)  36-45 years: 37 (37.0)  46-55 years: 24 (24.0)  > 55 years: 2 (2.0)  **Gender**  NR, presumably all male  **Response**  NR, but presumably 100% because the author was responsible for the occupational health screening which was mandatory in the German Democratic Republic.  **Exposure intensity**  Working as a hand welder (kneeling, squatting).  **Exposure duration**  NR, most of the time (not measured) kneeling or squatting work posture. | No controls were screened. | Meniscal lesions were assessed through clinical examination.  For the diagnosis of meniscal lesion three signs (pressure pain, Steinmann test, Krömer test) had to be positive in combination with atrophy of M. rectus femoris. |
| Nauwald (1986) | German Democratic Republic | Cross-sectional study | NR | Pipe-fitters in a shipbuilding plant.  n=101  **Age, years (mean)**  47.6  35-45 years (n (%)): 45 (44.6)  46-55 years (n (%)): 38 (37.6)  > 55 years (n (%)): 18 (17.8)  **Gender**  All male  **Response**  NR, but presumably 100% because the author was responsible for the occupational health screening which was mandatory in the German Democratic Republic.  **Exposure intensity**  Working as a pipe-fitter in a shipbuilding plant mostly in a stooped, kneeling or squatting posture.  **Exposure duration**  Mean duration of exposure: 25 years  Mean duration of exposure in different age groups:  35-45 years: 20.64 years  46-55 years: 27.57 years  > 55 years: 30.33 years | Persons without knee-straining exposure matched to age. The occupations of controls were not reported.  n=74  **Age**  NR, matched to exposure group  **Gender**  All male  **Response**  See left | Presence of meniscal lesions diagnosed clinically by unnamed meniscus tests. |
| Pressel (1982) | Federal Republic of Germany | Cross-sectional study | 1959-1975^1^ | Shunters from the German National Railway employed according the collective bargaining agreement and who were hired before 1975 and still active in 1976.  n=546  **Age, years (n (%))**  < 25: 87 (16)  25-34: 175 (32)  35-44: 197 (36)  45-54: 82 (15)  > 54: 6 (1)  **Gender**  All male  **Response**  NR, but presumably 100% because of the nature of the study as retrospective analysis of health insurance data.  **Exposure intensity**  Squatting (15.3 % of time), walking on slippery and uneven surfaces, running and jumping, climbing, kneeling, (stooping and lifting loads)    **Exposure duration, years (n (%))**  < 4: 235 (43)  4-5: 104 (19)  6-10: 98 (18)  11-20: 76 (14)  21-30: 27 (5)  > 30: 2 (0.4)  The percentages add up to 100.4 %. | Transport workers from the German National Railway.  n=532  **Age, years (n (%))**  < 24: 16 (3)  25-34: 74 (14)  35-44: 197 (37)  45-54: 176 (33)  > 54: 69 (13)  **Gender**  All male  **Response**  See left  **Exposure intensity**  Walking on even surfaces, stooping and lifting loads, e.g. weight of bags: 100 kg  **Exposure duration, years (n (%))**  < 4: 80 (15)  4-5: 90 (17)  6-10: 106 (20)  11-20: 170 (32)  21-30: 80 (15)  > 30: 3 (0.6)  The percentages add up to 99.6 %. | Suspicion of meniscopathy or probable meniscopathy according to medical records from the BBKK (health insurance of the German National Railway) registered between 1959 and 1975. The diagnosis of probable meniscopathy was based on surgical findings. |
| Prien et al. (2019) | Federal Republic of Germany | Cross-sectional study | NR | German former elite female football players, who had retired for at least 2 years.  n=49  **Age, years (mean, SD (range))**  37.2, 4.9 (30-47)  **Gender**  All female  **Response**  20%  **Exposure intensity**  Playing football in the first German football league and/or in the national team.  Trainings/week (mean, SD (range)): 5.0, 1.3 (3-8)  Matches/year (mean, SD (range)): 25.4, 12.4 (6-55)  **Exposure duration**  Career years (mean, SD (range)): 9.9, 5.3 (1-20) | No controls were screened. | Meniscus lesions diagnosed in 1.5-Tesla MRI according the classification of Stoller et al. (1987) with modifications: grade 1: horizontal/ intrasubstantial tear, grade 2: incomplete radial or oblique tear, grade 3: complex tear, grade 4: avulsed root or meniscal extrusion > 2 mm.  Comment: The classification used for meniscal lesions has nothing to do with the grading according to Stoller et al. (1987) which has only 3 grades and didn’t include complex tears and meniscus extrusions. |
| Roos et al. (1994) | Sweden | Cross-sectional study | 1988 | Former professional football players from two football clubs in Malmö.  n=71  **Age, years (mean)**  62.7  **Gender**  All male  **Response**  NR, but presumably 100% because of the nature of the study as retrospective analysis of hospital records.  **Exposure intensity**  Playing football on professional level in the first or second national league of Sweden.  **Exposure duration**  Participants played football on professional level at least until age 25. | Age-matched controls with unknown former football activity were selected in Malmö from the National Population Records.  n=142  **Age, years (mean)**  62.7  **Gender**  All male  **Response**  See left | Information on meniscectomies was retrieved from hospital register (General Hospital in Malmö - Department of Orthopaedics) from 1930-1989. |
| Rytter et al. (2008) | Denmark | Cross-sectional study | 2004-2006 | A sample of 286 male floor layers were established in 1994 based on trade union rosters. Workers, who were members of the trade union for floor layers  10 years earlier (1984) were also included. Members aged 36–70 years in 2004 and residents in Copenhagen and Aarhus, Denmark were included in the study.  n=134  **Age, years (mean, SD)**  52.6, 6.9  ≤ 49 (n (%)): 43 (32.1)  50-59 (n (%)): 72 (53.7)  ≥ 60 (n (%)): 19 (14.2)  **Gender**  All male  **Response**  Questionnaire: 88%  Clinical examination: 58%  **Exposure intensity**  Working as a floor layer  **Exposure duration**  Duration of employment, years (mean, SD): 29.2, 10.2 | A cohort of 370 graphic designers recruited in the same way as the floor layers (see left). Danish floor layers and graphic designers are comparable with respect to level of education and socio-economic status.  n=120  **Age, years (mean, SD)**  57.9, 5.9  ≤ 49 (n (%)): 7 (5.8)  50-59 (n (%)): 73 (60.8)  ≥ 60 (n (%)): 40 (33.4)  **Gender**  All male  **Response**  Questionnaire: 78%  Clinical examination: 46% | Possible meniscal lesions assessed through clinical knee examination (McMurray test and tibiofemoral joint line palpation).  A positive McMurray test was consistent with localized joint line pain or a palpable click bringing the knee from maximal flexion too extension, while the foot was retained in full external (medial meniscus) or internal (lateral meniscus) rotation. |
| Rytter et al. (2009); Jensen et al. (2012a); Jensen et al. (2012b) | Denmark | Cross-sectional study | 2004-2006 | A Danish sample of 286 male floor layers was established from trade union rosters comprising members aged 36–70 years in 2004. The workers were recruited in Copenhagen (capital city) and Aarhus (second largest city), Denmark.  n=92  **Age, years (mean, SD (range))**  54.5, 7.2 (42-70)  ≤49 (n (%)): 24 (26.1)  >50 (n (%)): 68 (73.9)  **Gender**  All male  **Response**  Questionnaire: 88%  MRI: 59%  **Exposure intensity**  Working as a floor layer  **Exposure duration**  Duration of employment, years (mean, SD): 29.6, 9.8  ≤20 (n (%)): 20 (21.8)  21-30 (n (%)): 27 (29.3)  31-35 (n (%)): 22 (23.9)  ≥36 (n (%)): 23 (25.0) | A cohort of 370 graphic designers recruited in the same way as the floor layers (see left). Danish floor layers and graphic designers are comparable with respect to level of education and socio-economic status.  n=49  **Age, years (mean, SD (range))**  57.7, 5.6 (42-70)  ≤49 (n (%)): 4 (8.2)  >50 (n (%)): 45 (91.8)  **Gender**  All male  **Response**  Questionnaire:78%  MRI: 32% | Grade 3 meniscal lesions according to Stoller et al. (1987) in a 1.5 Tesla MRI. |
| Walczak et al. (2008) | USA | Cross-sectional study | 2005 | Professional players in one club of the US-National Basketball Association screened prior to the start of the season.  n=14  **Age, years (mean (range))**  26.3 (20-36)  **Gender**  All male  **Response**  NR, presumably 100 % because of the nature of the study as preseason evaluation of professional players.  **Exposure intensity**  Playing basketball on professional level in the National Basketball Association (NBA).  **Exposure duration**  NR | No controls were screened. | Presence of meniscal lesions in both knees was assessed through 0.3-T magnet open extremity MRI, 0.7-T magnet open high field extremity MRI or 1.5-T MRI scanners.  Meniscal lesions were graded according to the system described by Zanetti et al. (2003).  Menisci were considered abnormal if there was intrameniscal high signal (MRI Grade II); a meniscus was considered torn if there was evidence of linear high signal extending to the articular surface (MRI Grade III). |

^1^ Investigation period in retrospective analyses

Study characteristics of included case-control studies

| Study | Study region | Time of recruitment | Population  *Source/setting, number of participants, age, gender* | | Exposure  *occupational exposure (definition, job title, duration, method used to identify exposure)* |
| --- | --- | --- | --- | --- | --- |
|  |  |  | Cases | Controls |  |
| Baker et al. (2002) | UK | March 1, 1996, to March 31, 1998 | Residents of Southampton and Portsmouth Health Districts, who were admitted to Southampton General  Hospital or Queen Alexandra Hospital, Portsmouth, during the 25-month period from March 1, 1996, to March 31, 1998, and in whom a diagnosis of meniscal tear was confirmed for the first time at arthroscopy.  n=243  **Age, years (mean (range))**  40.1 (20-59)  **Gender (n (%))**  Female: 47 (19.34)  Male: 196 (80.66)  **Response**  72% | Community controls of the same sex and matched as closely as possible for age, who were registered with the same general practitioner.  n=461  **Age, years**  NR  **Gender (n (%)**  NR  **Response**  28% | **Exposure duration/intensity**  Kneeling >1h/d, squatting >1h/d, lifting ≥10/20/50 kg >10 times/d, sport and other exposures assessed through a structured questionnaire. |
| Baker et al. (2003) | UK | NR | The study sample comprised a random one in six sample of men aged 20-59 years selected from the registration lists of 8 general practices in southern Hampshire. 1,404 completed a questionnaire. In a nested case-control study 67 male cases with meniscectomy were included.  n=67  **Age, years (mean (range))**  Age at which symptoms begun: 22 (8-44)  No information according the age at meniscectomy.  **Gender (n (%))**  All male  **Response**  50% of the original sample | For each man who reported undergoing meniscectomy five controls were randomly selected, matched to within one year of age, from the pool of subjects who had not undergone meniscectomy.  n=335  **Age, years**  Matched to cases  **Gender (n (%))**  All male  **Response**  See left | **Exposure duration/intensity**  Sitting for >2h, standing or walking for >2h, kneeling for >1h, squatting for >1h, getting up from kneeling or squatting >30 times, driving for >4 hours, walking for >2 miles, climbing >30 flights of stairs, lifting or moving weights of ≥10/≥25 kg by hand, work in an occupation likely to involve kneeling or squatting, sporting activities (soccer, rugby, running, swimming, other) assessed through a questionnaire. |
| Gotthardt et al. (1995); Gotthardt (1997) | Federal Republic of Germany | 1990-1993 | Patients, who were admitted to the surgical department of a German Hospital (Hamburg), between 1990 and 1993, and in whom a diagnosis of meniscopathy was confirmed surgically.  n=83  **Age, years (mean, SD)**  51, 14.6  **Gender (n (%))**  Female: 36 (43.4)  Male: 47 (56.6)  **Response**  97.6% | Patients admitted to the same hospital in the same period with a present disease in the mouth-jaw-face area (n=50) or abdominal diseases (n=27).  n=77  **Age, years (mean, SD)**  43, 19.5  **Gender (n (%))**  Female: 40 (51.9)  Male: 37 (48.1)  **Response**  91.7% | **Exposure intensity**  Kneeling, squatting, working on ladders, jumping activities, walking on uneven surface, lifting weights ≥50 kg, job title assessed through a standardized questionnaire.  **Exposure duration**  NR |
| Sharrard & Liddell (1962) | UK | January 1958 to June 1960^1^ | Patients between the ages of 15 and 64 with meniscectomy. Records were collected from five hospitals in one of the largest British coalfields.  n=957  **Age, years (range)**  15-64  **Gender**  All male  **Response**  NR, but presumably 100% because of the nature of the study as retrospective analysis of hospital records. | From the same hospitals the age and occupation of male patients with appendicectomy in the same age range during the same period were obtained.  n=1075  **Age, years**  NR  **Gender**  All male  **Response**  See left | **Exposure intensity**  Working as a hard coal miner (walking, standing, stooping; kneeling on heels; crawling or kneeling upright; kneeling with leg(s) rotated; sitting or squatting, without information according the duration of exposure)  **Exposure duration**  NR |

^1^ Investigation period in retrospective analyses
