# Supplementary material for: Occupational risk factors for meniscal lesions: a systematic review and meta-analysis
Source: BMC Musculoskelet Disord. 2021 Dec 15;22:1042. doi: 10.1186/s12891-021-04900-7 (PMC8672613; doi:10.1186/s12891-021-04900-7)
Supplement: Supplementary file 5 — Additional file 5. Results shown in included studies. [file 12891_2021_4900_MOESM5_ESM.docx]

**Additional file 5:** Results shown in included studies

Results shown in included cohort and cross-sectional studies

| Study | Prevalence or incidence of meniscal lesions (n/N (%) or n/person-years) | | | Risk estimate | | | Remarks |
| --- | --- | --- | --- | --- | --- | --- | --- |
|  | Effect estimate | Exposure group | Comparison group | Effect estimate | Effect value (95% CI) | Adjusted for |  |
| Behzadi et al. (2017) | Prevalence of |  |  | NR | Prevalence ratios should not be calculated because missing information according the response rate in controls. |  |  |
|  | Meniscal lesions | 1/22 (4.6) | 0/22 (0) |  |  |  |  |
|  | Meniscal degeneration | 12/22 (54.6) | 6/22 (27.3) |  |  |  |  |
|  | Grade II | 7/22 (31.8) | 4/22 (18.2) |  |  |  |  |
|  | Grade III | 5/22 (22.7) | 2/22 (9.1) |  |  |  |  |
| Bezuglov et al. (2019) | Prevalence of  Meniscal lesion – horn of the lateral meniscus  Anterior: |  | No controls screened. | NR |  |  | In 93.6% of the examined soccer players, both joints were affected by meniscal lesions. In 4.2%, meniscal lesions were detected in 1 joint only, and 2.2% were unaffected. Overall, 27.7% of the participants had 1 to 3 lesions; 40.4% had 4 to 7 lesions; and 31.9% had 8 lesions, with grade 2 meniscal lesions being predominant. The posterior horn of the lateral meniscus was the most frequent lesion site (95.7% of all the joints) |
|  | Absent | 42^1^/94 (44.7) |  |  |  |  |  |
|  | Grade I | 12^1^/94 (12.8) |  |  |  |  |  |
|  | Grade II | 36^1^/94 (38.3) |  |  |  |  |  |
|  | Grade III | 4^1^/94 (4.3) |  |  |  |  |  |
|  | Posterior: |  |  |  |  |  |  |
|  | Absent | 35^1^/94 (37.2) |  |  |  |  |  |
|  | Grade I | 12^1^/94 (12.8) |  |  |  |  |  |
|  | Grade II | 46^1^/94 (48.9) |  |  |  |  |  |
|  | Grade III | 1^1^/94 (1.1) |  |  |  |  |  |
|  | Meniscal lesion – horn of the medial meniscus  Anterior: |  |  |  |  |  |  |
|  | Absent | 35^1^/94 (37.2) |  |  |  |  |  |
|  | Grade I | 17^1^/94 (18.1) |  |  |  |  |  |
|  | Grade II | 41^1^/94(43.6) |  |  |  |  |  |
|  | Grade III | 1^1^/94 (1.1) |  |  |  |  |  |
|  | Posterior: |  |  |  |  |  |  |
|  | Absent | 4^1^/94 (4.3) |  |  |  |  |  |
|  | Grade I | 16^1^/94 (17.0) |  |  |  |  |  |
|  | Grade II | 67^1^/94 (71.3) |  |  |  |  |  |
|  | Grade III | 7^1^/94 (7.4) |  |  |  |  |  |
| Brouwer et al. (1981) | Prevalence of  Meniscectomy | 14/43 (32.6) | NR |  |  |  |  |
|  | Medial | 4/43 (9.3) |  |  |  |  |  |
|  | Lateral | 3/43 (7) |  |  |  |  |  |
|  | Bilateral | 7/43 (16.3) |  |  |  |  |  |
| Hong et al. (2020) | Prevalence of  Meniscal tears | 265/486 (54.5) |  | OR |  | Gender, age, BMI, mechanical axis, knee injury history, cumulative squatting working time/cumulative heavy-lifting working time |  |
|  | Female | 149/248 (60.1) |  |  |  |  |  |
|  | Medial | 134/248 (54.0) |  |  |  |  |  |
|  | Lateral | 62/248 (25.0) |  |  |  |  |  |
|  | Male | 116/238 (48.7) |  |  |  |  |  |
|  | Medial | 105/238 (44.1) |  |  |  |  |  |
|  | Lateral | 33/238 (13.9) |  |  |  |  |  |
|  | Meniscal tears  Cumulative heavy-lifting working time (hours) |  |  |  |  |  |  |
|  | <2,000 |  | 81/156 (51.9) |  | 1.00 (ref) |  |  |
|  | 2,000-4,999 | 32/59 (54.2) |  |  | 0.99 (0.51–1.94) |  |  |
|  | ≥5,000 | 152/271 (56.1) |  |  | 0.76 (0.47–1.24) |  |  |
|  | Cumulative squatting working time (hours) |  |  |  |  |  |  |
|  | <10,000 |  | 106/238 (44.5) |  | 1.00 (ref) |  |  |
|  | 10,000-19,999 | 37/64 (57.8) |  |  | 2.16 (1.14–4.07) |  |  |
|  | ≥20,000 | 122/184 (66.3) |  |  | 2.35 (1.45–3.80) |  |  |
| Kaplan et al. (2005) | Prevalence of  Meniscal tears | 8/40 (20) | No controls screened. | NR |  |  |  |
|  | Medial | 7/40 (17.5) |  |  |  |  |  |
|  | Lateral | 1/40 (2.5) |  |  |  |  |  |
|  | Jumping knee  Medial meniscus  Posterior horn |  |  |  |  |  |  |
|  | Grade 1 | 4/20 (20) |  |  |  |  |  |
|  | Grade 2 | 0/20 (0) |  |  |  |  |  |
|  | Grade 3 | 0/20 (0) |  |  |  |  |  |
|  | Medial body |  |  |  |  |  |  |
|  | Grade 1 | 0/20 (0) |  |  |  |  |  |
|  | Grade 2 | 0/20 (0) |  |  |  |  |  |
|  | Grade 3 | 0/20 (0) |  |  |  |  |  |
|  | Anterior horn |  |  |  |  |  |  |
|  | Grade 1 | 0/20 (0) |  |  |  |  |  |
|  | Grade 2 | 0/20 (0) |  |  |  |  |  |
|  | Grade 3 | 0/20 (0) |  |  |  |  |  |
|  | Lateral meniscus  Posterior horn |  |  |  |  |  |  |
|  | Grade 1 | 0/20 (0) |  |  |  |  |  |
|  | Grade 2 | 0/20 (0) |  |  |  |  |  |
|  | Grade 3 | 0/20 (0) |  |  |  |  |  |
|  | Lateral body |  |  |  |  |  |  |
|  | Grade 1 | 0/20 (0) |  |  |  |  |  |
|  | Grade 2 | 0/20 (0) |  |  |  |  |  |
|  | Grade 3 | 0/20 (0) |  |  |  |  |  |
|  | Anterior horn |  |  |  |  |  |  |
|  | Grade 1 | 0/20 (0) |  |  |  |  |  |
|  | Grade 2 | 0/20 (0) |  |  |  |  |  |
|  | Grade 3 | 0/20 (0) |  |  |  |  |  |
|  | Nonjumping knee  Medial meniscus  Posterior horn |  |  |  |  |  |  |
|  | Grade 1 | 2/20 (10) |  |  |  |  |  |
|  | Grade 2 | 0/20 (0) |  |  |  |  |  |
|  | Grade 3 | 1/20 (5) |  |  |  |  |  |
|  | Anterior horn |  |  |  |  |  |  |
|  | Grade 1 | 0/20 (0) |  |  |  |  |  |
|  | Grade 2 | 0/20 (0) |  |  |  |  |  |
|  | Grade 3 | 0/20 (0) |  |  |  |  |  |
|  | Lateral meniscus Posterior horn |  |  |  |  |  |  |
|  | Grade 1 | 0/20 (0) |  |  |  |  |  |
|  | Grade 2 | 0/20 (0) |  |  |  |  |  |
|  | Grade 3 | 0/20 (0) |  |  |  |  |  |
|  | Lateral body |  |  |  |  |  |  |
|  | Grade 1 | 0/20 (0) |  |  |  |  |  |
|  | Grade 2 | 0/20 (0) |  |  |  |  |  |
|  | Grade 3 | 1/20 (5) |  |  |  |  |  |
|  | Anterior horn |  |  |  |  |  |  |
|  | Grade 1 | 0/20 (0) |  |  |  |  |  |
|  | Grade 2 | 0/20 (0) |  |  |  |  |  |
|  | Grade 3 | 0/20 (0) |  |  |  |  |  |
| Kivimäki et al. (1992) | Prevalence of  Meniscus rupture | 17^1^/168 (10.1)^1^ | 7^1^/146 (4.8)^1^ | Prevalence ratio^1^ | 2.11 (0.90–4.95) | None (but all workers were male and frequency matched according to five-year strata). |  |
| Kontio et al. (2017) | Incidence of  Hospitalization due to meniscal lesions  Physical strenuousness of work |  |  | HR |  | Age, gender, education, BMI, leisure time physical exercise, smoking, alcohol intake |  |
|  | Light |  | 65/1264 (5.1) |  | 1.00 (ref) |  |  |
|  | Moderate | 115/1954 (5.9) |  |  | 1.20 (0.86–1.64) |  |  |
|  | Heavy | 35/1043 (3.4) |  |  | 0.77 (0.48–1.20) |  |  |
| Krajnc et al. (2010) | Prevalence of  Total meniscus surgeries | 20/40 (50.0) | No controls screened. | NR |  |  |  |
|  | Isolated meniscus surgeries | 13/40 (32.5) |  |  |  |  |  |
|  | Meniscus + ACL | 5/40 (12.5) |  |  |  |  |  |
|  | Meniscus + collateral ligament | 2/40 (5.0) |  |  |  |  |  |
| Mikkelsen et al. (2016) | Incidence of  Meniscal tears  Incidence rate per 10,000 person-years | 277/3,307 (8.4)  62.13 | 3,243/63,934 (5.1)  34.15 | Adjusted incidence rate ratio of  Baggage handlers | 1.00 (ref) | Age, calendar year, use of baggage lifter, use of baggage belt loader, educational level and pre-employment knee injuries | In the analysis stratified by work area only work as baggage handler on the apron showed a relationship with meniscopathy risk. The study cannot differentiate between medial and lateral meniscus lesion. Therefor the risk of baggage handlers for medial meniscus lesions may be underestimated. |
|  |  |  |  | Controls | 0.64 (0.52–0.80) |  |  |
|  |  |  |  | Baggage handler years (categorical)  Controls | 0.79 (0.60–1.04) |  |  |
|  |  |  |  | 0.1-2.9 years | 1.00 (ref) |  |  |
|  |  |  |  | 3.0-9.9 years | 1.38 (1.04–1.83 |  |  |
|  |  |  |  | 10-19.9 years | 1.40 (1.01–1.94) |  |  |
|  |  |  |  | ≥20 years | 1.02 (0.61–1.71) |  |  |
|  |  |  |  | Baggage handler years (linear)  Controls | 0.66 (0.52–0.83) |  |  |
|  |  |  |  | Baggage handlers | 1.00 (ref) |  |  |
|  |  |  |  | Continuous linear (per 5 years) | 1.01 (0.93–1.10) |  |  |
|  |  |  |  | Baggage handler years (linear spline)  Controls | 0.95 (0.68–1.34) | Also adjusted for baggage handler (yes/no) |  |
|  |  |  |  | Baggage handlers | 1.00 (ref) |  |  |
|  |  |  |  | Continuous linear 0-4.9 years (per 5 years) | 1.91 (1.29–2.84) |  |  |
|  |  |  |  | Continuous linear ≥5 years (per 5 years) | 0.88 (0.78–1.00) |  |  |
| Musialek & Kostal (1995) | Prevalence of  Meniscus lesions treated with open surgery per 10,000 per year | 98/19,170 in 2 years (25.6) | 192/310,000 in 2 years (3.1) | Prevalence ratio^1^ | 8.25 (6.47–10.52) |  | The authors reported incidences of open surgeries, but to our assessment this is no cohort study. Furthermore, they reported an incidence of 6.2 per 10,000 per year in controls. This is to our opinion a calculation error. |
|  | Medial meniscal lesion | 83^1^/98 (84.6) | 170^1^/192 (88.5) |  |  |  |  |
|  | Lateral meniscal lesion | 15^1^/98 (15.4) | 22^1^/192 (11.5) |  |  |  |  |
|  | Lesion of posterior horn | 32^1^/98 (32.6) | 48^1^/192 (24.9) |  |  |  |  |
|  | Lesion of anterior horn | 66^1^/98 (67.4) | 144^1^/192 (75.1) |  |  |  |  |
| Nauwald (1980) | Prevalence of  Meniscal damage  Right knee | 19/100 (19) | No controls screened. | NR |  |  | The figure shows a prevalence of 12 meniscal lesions in the left knee, but according to the table there are 17 meniscal lesions. |
|  | ≤ 25 years | 1^1^/9 (11.1) |  |  |  |  |  |
|  | 26-35 years | 4^1^/28 (14.3) |  |  |  |  |  |
|  | 36-45 years | 8^1^/37 (21.6) |  |  |  |  |  |
|  | 46-55 years | 5^1^/24 (20.8) |  |  |  |  |  |
|  | > 55 years | 1^1^/2 (50.0) |  |  |  |  |  |
|  | Left knee | 17/100 (17) |  |  |  |  |  |
|  | ≤ 25 years | 1^1^/9 (11.1) |  |  |  |  |  |
|  | 26-35 years | 6^1^/28 (21.4) |  |  |  |  |  |
|  | 36-45 years | 5^1^/37 (13.5) |  |  |  |  |  |
|  | 46-55 years | 3^1^/24 (12.5) |  |  |  |  |  |
|  | > 55 years | 2^1^/2 (100.0) |  |  |  |  |  |
| Nauwald (1986) | Prevalence of  Meniscal lesions  Right knee | 12/101 (11.8) | 0/74 (0) | Prevalence ratio^1, 2^ |  |  |  |
|  | Left knee | 7/101 (6.9) | 0/74 (0) |  | 11.03 (0.64–190.13) |  |  |
| Pressel (1980) | Prevalence of  Suspicious of meniscal lesion | 3/546 (0.5) | 7/532 (1.3) |  |  |  |  |
|  | Possible meniscal lesion | 2/546 (0. 4) | 2/532 (0.4) |  |  |  |  |
| Prien et al. (2019) | Prevalence of  Substantial meniscal loss (meniscus lesions ≥ 3 or meniscus extrusion > 2 mm) | 34/49 (69.4) | No controls screened. | OR for meniscal loss  Traumatic injury  No | 1.00 (ref) | Age, BMI |  |
|  | Meniscal lesion  Medial meniscus | 32/98 (32.7) |  | Yes | 2.64 (1.14–6.10) |  |  |
|  |  |  |  | Injured leg  No injury | 1.00 (ref) |  |  |
|  | Lateral meniscus | 32/98 (32.7) |  |  |  |  |  |
|  | Meniscus extrusion  Medial meniscus | 24/98 (24.5) |  | Non-striking leg | 10.59 (3.18–35.28) |  |  |
|  | Lateral meniscus | 25/98 (25.5) |  | Striking leg | 2.66 (0.89–7.86) |  |  |
|  |  |  |  | Type of injury  No injury | 1.00 (ref) |  |  |
|  |  |  |  | ACL | 3.55 (0.93–13.55) |  |  |
|  |  |  |  | Meniscus | 3.42 (0.96–12.23) |  |  |
|  |  |  |  | ACL + meniscus | 9.47 (2.38–37.62) |  |  |
|  |  |  |  | Other injury | 5.28 (1.40–19.96) |  |  |
|  |  |  |  | Age meniscus/ ACL injury | 0.78 (0.63–0.97) |  |  |
| Roos et al. (1994) | Prevalence of  Meniscectomies 1930-1989 | 12/71 (16.9) | 3/142 (2.1) | Prevalence ratio^1^ | 8.0 (2.33–27.44) | No adjustment, football players and controls are all male and controls are age-matched to the football players. | In 71.7% the medial meniscus was operated. |
| Rytter et al. (2008) | Prevalence of  Positive McMurray test | 32/134 (23.9) | 14/120 (11.7) | OR | 2.4 (1.1–5.0) | Age, BMI, knee straining sports |  |
|  | After exclusion of floor layers (n=22) and graphic designers (n=15) with radiographic tibiofemoral osteoarthritis | NR | NR |  | 2.2 (1.0–4.9) |  |  |
|  | Tenderness on palpating the tibiofemoral joint line | 42/134 (31.32) | 11/120 (9.2) |  | 5.4 (2.4–12.0) |  |  |
|  | After exclusion of floor layers (n=22) and graphic designers (n=15) with radiographic tibiofemoral osteoarthritis | NR | NR |  | 5.0 (2.0–12.5) |  |  |
| Rytter et al. (2009) | Prevalence of  Grade III medial meniscus lesion | 62/92 (67.4) | 26/49 (53.1) | OR | 2.28 (1.10–4.98) | Age, BMI, sports |  |
|  | Unilateral | 15/92 (16.3) | 11/49 (22.4) |  | 0.95 (0.33–2.67) |  |  |
|  | Bilateral | 47/92 (51.1) | 15/49 (30.6) |  | 3.46 (1.41–8.48) |  |  |
|  | Grade III lateral meniscus lesion | 12/92 (13.00) | 11/49 (22.4) |  | 0.75 (0.29–1.98) |  |  |
|  | Unilateral | 11/92 (12.0) | 9/49 (18.4) |  | 0.78 (0.28–2.17) |  |  |
|  | Bilateral | 1/92 (1.1) | 2/49 (4.1) |  | 0.50 (0.04–6.51) |  |  |
|  | Grade III medial meniscus lesion stratified into age groups  ≤49 years | 16/24 (66.7) | 2/4 (50.0) |  | 2.0 (0.1–34.3) | BMI, trade seniority, sports |  |
|  | 50-59 years | 29/45 (64.4) | 13/28 (46.4) |  | 2.7 (0.9–8.2) |  |  |
|  | ≥60 years | 17/23 (73.9) | 11/17 (64.7) |  | 2.5 (0.4–17.0) |  |  |
| Jensen et al. (2012a) | Prevalence of  Symptomatic medial meniscal tear | 26/85 (30.6) | 7/40 (17.5) | OR | 2.04 (0.77–5.5) | Age, BMI, sports, and traumas |  |
|  | Meniscal tears | 68/85 (80.0) | 27/40 (67.5) |  | NR |  |  |
|  | Medial | 56/85 (65.9) | 19/40 (47.5) |  | NR |  |  |
|  | Lateral | 12/85 (14.1) | 8/40 (20.0) |  | NR |  |  |
| Jensen et al. (2012b) | Prevalence of  Grade III medial meniscus lesion (ever exposed) | 62/92 (67.4) | 26/49 (53.1) | OR | 2.82 (1.25–2.10) | Age, BMI, knee straining sport activities, previous knee traumas |  |
|  | ≤20 years | NR | NR |  | 4.73 (1.16–19.4) |  |  |
|  | 21-30 years | NR | NR |  | 4.59 (1.28–16.5) |  |  |
|  | >30 years | NR | NR |  | 1.96 (0.79–4.88) |  |  |
|  | Grade III lateral meniscus lesion (ever exposed) | 12/92 (13.0) | 11/49 (22.4) |  | 0.78 (0.29–2.10) |  |  |
|  | ≤20 years | NR | NR |  | 1.14 (0.24–5.33) |  |  |
|  | 21-30 years | NR | NR |  | 0.68 (0.11–4.28) |  |  |
|  | >30 years | NR | NR |  | 0.86 (0.27–2.76) |  |  |
| Walczak et al. (2008) | Prevalence of  Degenerative meniscus changes (grade II) | 15/28 (53.6) | No controls screened. |  |  |  |  |
|  | Right knee | 8/14 (57.1) |  |  |  |  |  |
|  | Left knee | 7/14 (50.0) |  |  |  |  |  |
|  | Possible meniscal tear | 2/28 (7.1) |  |  |  |  |  |
|  | Right knee | 2/14 (14.3) |  |  |  |  |  |
|  | Left Knee | 0/14 (0.0) |  |  |  |  |  |
|  | Meniscal tear (grade III) | 1/28 (3.6) |  |  |  |  |  |
|  | Right knee | 0/14 (0.0) |  |  |  |  |  |
|  | Left knee | 1/14 (7.1) |  |  |  |  |  |

ACL: Anterior cruciate ligament; BMI: Body Mass Index; CI: Confidence interval; HR: Hazard ratio; NR: Not reported; OR: Odds ratio

^1^ Calculated by the authors of this review

^2^ To allow the calculation of a prevalence ratio a prevalence of 0.5 % in controls was assumed.

Results shown in included case-control studies

| Study | Exposure | Exposed cases (n/N (%)) | Exposed controls (n/N (%)) | Risk estimate | | | Remarks |
| --- | --- | --- | --- | --- | --- | --- | --- |
|  |  |  |  | Effect estimate | Effect value (95% CI) | Adjusted for |  |
| Baker et al. (2002) | Kneeling > 1 h in total per day | 68/202 (33.7) | 67/333 (20.1) | OR | 2.2 (1.3–3.6) | BMI, Beighton score,  participation in sports, social status | *No distinction between professional and amateur athletes.  Probably mainly hobby sportsmen because of the high prevalence |
|  | Squatting > 1 h in total per day | 61/202 (30.2) | 62/333 (18.6) |  | 1.8 (1.1–3.0) |  |  |
|  | Getting up from kneeling or  squatting > 30 times per day | 95/202 (47.0) | 101/333 (30.3) |  | 1.9 (1.2–3.1) |  |  |
|  | Climbing > 30 flights of stairs per day | 102/202 (50.5) | 94/333 (28.2) |  | 2.4 (1.6–3.8) |  |  |
|  | Lifting or carrying ≥10 kg, > 10 times per week | 125/202 (61.9) | 146/333 (43.8) |  | 1.9 (1.2–2.9) |  |  |
|  | Lifting or carrying ≥25 kg, > 10 times per week | 78/202 (38.6) | 88/333 (26.4) |  | 1.7 (1.1–2.7) |  |  |
|  | Lifting or carrying ≥50 kg, > 10 times per week | 50/202 (24.8) | 36/333 (10.8) |  | 2.4 (1.4–4.2) |  |  |
|  | Walking > 2 miles in total per day | 140/202 (69.3) | 185/333 (55.6) |  | 1.5 (0.9–2.3) |  |  |
|  | Standing or walking > 2 h in total per day | 161/202 (79.7) | 231/333 (69.4) |  | 1.3 (0.8–2.2) |  |  |
|  | Driving > 4 hours in total per day | 52/202 (25.7) | 63/333 (18.9) |  | 2.3 (1.4–4.0) |  |  |
|  | Sitting > 2 h in total per day | 112/202 (55.4) | 220/333 (66.1) |  | 0.9 (0.6–1.4) |  |  |
|  | Occupational or hobby participation in sport* | | |  |  |  |  |
|  | Soccer | 61/202 (30.2) | 47/333 (14.1) |  | 3.7 (2.1–6.6) |  |  |
|  | Rugby | 7/202 (3.5) | 5/333 (1.5) |  | 2.2 (0.6–8.2) |  |  |
|  | Running | 19/202 (9.4) | 27/333 (8.1) |  | 1.4 (0.7–2.8) |  |  |
|  | Swimming | 58/202 (28.7) | 70/333 (21.0) |  | 1.6 (1.0–2.5) |  |  |
| Baker et al. (2003) | Kneeling for >1 hour in total | 23/67 (34.3) | 56/335 (16.7) | OR | 2.5 (1.3–4.8) | Participation in sports | *No distinction between professional and amateur athletes.  Probably mainly hobby sportsmen because of the high prevalence |
|  | Squatting for >1 hour in total | 20/67 (29.9) | 47/335 (14.0) |  | 2.5 (1.2–4.9) |  |  |
|  | Getting up from kneeling or squatting >30 times | 23/67 (34.3) | 76/335 (22.7) |  | 1.9 (1.0–3.8) |  |  |
|  | Climbing >30 flights of stairs | 18/67 (26.9) | 59/335 (17.6) |  | 2.0 (1.0–4.1) |  |  |
|  | Lifting or moving weights of ≥10 kg by hand | 31/67 (46.3) | 120/335 (35.8) |  | 1.7 (0.9–3.1) |  |  |
|  | Lifting or moving weights of ≥25 kg by hand | 20/67 (29.9) | 86/335 (25.7) |  | 1.3 (0.7–2.5) |  |  |
|  | Work in an occupation likely to involve kneeling or squatting | 17/67 (25.4) | 61/335 (18.2) |  | 2.3 (1.1–4.8) |  |  |
|  | Walking for >2 miles in total | 25/67 (37.3) | 102/335 (30.4) |  | 1.1 (0.6–2.2) |  |  |
|  | Standing or walking for >2 hours in total | 43/67 (64.2) | 184/335 (54.9) |  | 1.5 (0.8–3.1) |  |  |
|  | Driving for >4 hours in total | 8/67 (11.9) | 40/335 (11.9) |  | 0.9 (0.4–2.1) |  |  |
|  | Sitting for >2 hours in total | 26/67 (38.8) | 150/335 (44.8) |  | 0.8 (0.4–1.6) |  |  |
|  | Occupational or hobby participation in sport* | | |  |  |  |  |
|  | Soccer | 36/67 (53.7) | 62/335 (18.5) |  | 6.9 (3.5–13.3) |  |  |
|  | Rugby | 11/67 (16.4) | 20/335 (6.0) |  | 3.4 (1.5–7.8) |  |  |
|  | Running | 6/67 (9.0) | 22/335 (6.6) |  | 1.4 (0.5–3.7) |  |  |
|  | Swimming | 14/67 (20.9) | 47/335 (14.0) |  | 1.6 (0.8–3.0) |  |  |
| Gotthardt et al. (1995); Gotthardt (1997) | Job title^1^ | | | OR |  | Age, sex, participation in sports, axial deviations of the knee, overweight | Medial meniscal lesion: 59/83 (71.1)  Lateral meniscal lesion: 14/83 (16.9) |
|  | Pipe fitter and installers | 6/83 (7.2) | 2/77 (2.6) |  | 0.88 (0.46–13.48) |  |  |
|  | Metal processing workers | 15/83 (18.1) | 9/77 (11.7) |  | 0.88 (0.31–2.51) |  |  |
|  | Trade and administration occupations | 54/83 (65.1) | 41/77 (53.2) |  | 2.11 (0.92–4.83) |  |  |
|  | Precision engineering occupations | 10/83 (12.0) | 6/77 (7.8) |  | 1.51 (0.42–5.42) |  |  |
|  | Textile and leather workers | 6/83 (7.2) | 4/77 (5.2) |  | 0.71 (0.16–3.20) |  |  |
|  | Construction workers | 16/83 (19.3) | 12/77 (15.6) |  | 0.81 (0.31-2.15) |  |  |
|  | Health professions | 37/83 (44.6) | 30/77 (39.0) |  | 1.00 (0.46–2.17) |  |  |
|  | Food and beverage workers | 8/83 (9.6) | 7/77 (9.1) |  | 0.70 (0.21–2.30) |  |  |
|  | Transport occupations | 16/83 (19.3) | 14/77 (18.2) |  | 0.77 (0.31–1.95) |  |  |
|  | Miners | 1/83 (1.2) | 1/77 (1.3) |  | 0.48 (0.04–5.91) |  |  |
|  | Paper and printing workers | 3/83 (3.6) | 3/77 (3.9) |  | 1.82 (0.27–12.39) |  |  |
|  | Others | 6/83 (7.2) | 15/77 (19.5) |  | 0.61 (0.17–2.16) |  |  |
|  | Stone and soil industry occupations | 3/83 (3.6) | 0/77 |  | Not calculable |  |  |
|  | Occupational activities | | |  |  | Age, sex, participation in sports, axial deviations of the knee, overweight |  |
|  | Working on ladders | 47/83 (56.6) | 28/77 (36.4) |  | 0.97 (0.54–1.74) |  |  |
|  | Kneeling | 48/83 (57.8) | 30/77 (39.0) |  | 1.72 (0.24–12.55) |  |  |
|  | Shock-like exposure, e.g. jumping | 31/83 (37.3) | 16/77 (20.8) |  | 1.11 (0.81-1.52) |  |  |
|  | Squatting | 45/83 (54.2) | 33/77 (42.9) |  | 1.39 (0.44-4.34) |  |  |
|  | Lifting weights ≥50 kg | 49/83 (59.0) | 37/77 (48.1) |  | 0.93 (0.59-1.46) |  |  |
|  | Walking on uneven surfaces | 42/83 (50.6) | 32/77 (41.6) |  | 1.13 (0.84-1.52) |  |  |
|  | 1-3 occupational activities | 35/83 (42.2) | 26/77 (33.8) |  | 2.59 (1.12–6.00) | / |  |
|  | 4-6 occupational activities | 35/83 (42.2) | 26/77 (33.8) |  | 2.59 (1.12–6.00) |  |  |
|  | Duration of occupational activities (≤ median) | | |  |  | / |  |
|  | Working on ladders  (0.22 years)^2^ | 16/83 (19.3) | 8/77 (10.4) |  | 2.31 (0.91–5.84) |  |  |
|  | Kneeling (0.47 years)^2^ | 20/83 (24.1) | 18/77 (23.4) |  | 1.41 (0.66–3.01) |  |  |
|  | Shock-like exposure, e.g. jumping (11.28 years)^2^ | 13/83 (15.7) | 10/77 (13.0) |  | 1.41 (0.57–3.47) |  |  |
|  | Squatting (0.32 years)^2^ | 18/83 (21.7) | 19/77 (24.7) |  | 1.03 (0.48–2.20) |  |  |
|  | Lifting weights ≥50 kg (0.69 years)^2^ | 15/83 (18.1) | 15/77 (19.5) |  | 1.02 (0.45–2.29) |  |  |
|  | Walking on uneven surfaces (1.02 years)^2^ | 19/83 (22.9) | 18/77 (23.4) |  | 1.10 (0.51–2.36) |  |  |
|  | Duration of occupational activities (> median) | | |  |  | / |  |
|  | Working on ladders  (0.22 years)^1,2^ | 16/83 (19.3) | 10/77 (13.0) |  | 1.85 (0.77–4.44) |  |  |
|  | Kneeling (0.47 years)^1,2^ | 22/83 (26.5) | 7/77 (9.1) |  | 3.98 (1.55–10.23) |  |  |
|  | Shock-like exposure, e.g. jumping (11.28 years)^1,2^ | 19/83 (22.9) | 12/77 (15.6) |  | 2.83 (0.95–8.44) |  |  |
|  | Squatting (0.32 years)^1,2^ | 18/83 (21.7) | 7/77 (9.1) |  | 2.79 (1.07–7.28) |  |  |
|  | Lifting weights ≥50 kg (0.69 years)^1,2^ | 15/83 (18.1) | 8/77 (10.4) |  | 1.91 (0.75–4.88) |  |  |
|  | Walking on uneven surfaces (1.02 years)^1,2^ | 13/83 (15.7) | 5/77 (6.5) |  | 1.65 (0.72–3.79) |  |  |
|  | Latency of occupational activities (≤ median) | | |  |  | / |  |
|  | Working on ladders  (32.5 years) ^1,2^ | 23/83 (33.7) | 18/77 (23.4) |  | 1.69 (0.80–3.58) |  |  |
|  | Kneeling (34.5 years)^1,2^ | 24/83 (28.9) | 18/77 (23.4) |  | 1.79 (0.84–3.80) |  |  |
|  | Shock-like exposure, e.g. jumping (22.0 years)^1,2^ | 16/83 (19.3) | 12/77 (15.6) |  | 1.56 (0.68–4.95) |  |  |
|  | Squatting (28.5 years)^1,2^ | 22/83 (26.5) | 19/77 (24.7) |  | 1.31 (0.62–2.77) |  |  |
|  | Lifting weights ≥50 kg (34.0 years)^1,2^ | 25/83 (30.1) | 24/77 (31.2) |  | 1.19 (0.58–2.46) |  |  |
|  | Walking on uneven surfaces (41.0 years)^1,2^ | 22/83 (26.5) | 27/77 (35.1) |  | 0.85 (0.42–5.09) |  |  |
|  | Latency of occupational activities (> median) | | |  |  | / |  |
|  | Working on ladders  (32.5 years)^1,2^ | 23/83 (27.7) | 10/77 (13.0) |  | 3.05 (1.29–4.76) |  |  |
|  | Kneeling (34.5 years)^1,2^ | 24/83 (28.9) | 12/77 (15.6) |  | 2.69 (1.19–6.11) |  |  |
|  | Shock-like exposure, e.g. jumping (22.0 years)^1,2^ | 15/83 (18.1) | 4/77 (5.2) |  | 4.40 (1.37–14.08) |  |  |
|  | Squatting (28.5 years)^1,2^ | 22/83 (26.5) | 14/77 (18.2) |  | 1.77 (0.80–3.93) |  |  |
|  | Lifting weights ≥50 kg (34.0 years)^1,2^ | 24/83 (28.9) | 14/77 (18.2) |  | 1.97 (0.88–4.40) |  |  |
|  | Walking on uneven surfaces (41.0 years)^1,2^ | 19/83 (22.9) | 6/77 (7.8) |  | 3.32 (1.21–9.12) |  |  |
| Sharrard & Liddell (1962) | Work as a hard coal miner | 605/957 (63.2) | 365/1075 (34.0) | OR  All age groups | 3.34^1^ (2.79–4.01) | No CI is given for the OR in the different age groups. |  |
|  |  |  |  | Age 15-24 | 1.5–5.0 |  |  |
|  |  |  |  | Age 25-34 | 3.0–13.5 |  |  |
|  |  |  |  | Age 35-44 | 3.5–22.7 |  |  |
|  |  |  |  | Age 45-54 | 1.2–5.3 |  |  |
|  |  |  |  | Age 55-64 | 1.6–2.2 |  |  |

BMI: Body Mass Index; CI: Confidence interval; OR: Odds ratio

^1^ Median

^2^ Ever exposed, multiple exposures possible
